# The current state of genetic risk models for the development of kidney cancer: a review and validation

**DOI:** 10.1111/bju.15752

**Published:** 2022-05-07

**Authors:** Hannah Harrison, Nicole Li, Catherine L. Saunders, Sabrina H. Rossi, Joe Dennis, Simon J. Griffin, Grant D. Stewart, Juliet A. Usher‐Smith

**Affiliations:** ^1^ Department of Public Health and Primary Care University of Cambridge Cambridge UK; ^2^ Deanary of Biomedical Sciences University of Edinburgh Edinburgh UK; ^3^ Department of Surgery University of Cambridge Addenbrooke’s Hospital Cambridge UK

**Keywords:** RCC, kidney cancer, genetics, risk models, risk stratification, polygenic risk scores, validation

## Abstract

**Objective:**

To review the current state of genetic risk models for predicting the development of kidney cancer, by identifying and comparing the performance of published models.

**Methods:**

Risk models were identified from a recent systematic review and the Cancer‐PRS web directory. A narrative synthesis of the models, previous validation studies and related genome‐wide association studies (GWAS) was carried out. The discrimination and calibration of the identified models was then assessed and compared in the UK Biobank (UKB) cohort (cases, 452; controls, 487 925).

**Results:**

A total of 39 genetic models predicting the development of kidney cancer were identified and 31 were validated in the UKB. Several of the genetic‐only models (seven of 25) and most of the mixed genetic‐phenotypic models (five of six) had some discriminatory ability (area under the receiver operating characteristic curve >0.5) in this cohort. In general, models containing a larger number of genetic variants identified in GWAS performed better than models containing a small number of variants associated with known causal pathways. However, the performance of the included models was consistently poorer than genetic risk models for other cancers.

**Conclusions:**

Although there is potential for genetic models to identify those at highest risk of developing kidney cancer, their performance is poorer than the best genetic risk models for other cancers. This may be due to the comparatively small number of genetic variants associated with kidney cancer identified in GWAS to date. The development of improved genetic risk models for kidney cancer is dependent on the identification of more variants associated with this disease. Whether these will have utility within future kidney cancer screening pathways is yet to determined.

## Background

Recent developments in genetic research have led to the identification of 100s of genetic variants associated with the development of different cancers [1]. Advances in sequencing technologies mean it is now possible to obtain genetic information from individuals at relatively low cost ($35 per individual [2]). Therefore, there is potential for genetic risk models, including polygenic risk scores (PRSs) that combine multiple single nucleotide polymorphisms (SNPs) together to estimate the risk of a disease or disease‐related trait for an individual, to enhance risk prediction and improve the efficiency of population‐level screening for cancer [2]. The Breast and Ovarian Analysis of Disease and Carrier Estimation Algorithm (BODICEA) model for breast cancer, for example, which includes 313 SNPs alongside phenotypic risk factors, is already used to support clinical decision‐making [2, 3] and studies are on‐going to evaluate the role of this model within screening programmes [4, 5].

There are several features of genetic risk models that will appeal to both clinicians and researchers. Firstly, germline genetic risk factors, including SNPs, do not change over the lifetime of an individual. This facilitates lifetime risk prediction rather than fixed‐time risk predictions (e.g., the 5‐ or 10‐year risk) and may help identify younger individuals at higher risk before the development of other risk factors. Secondly, genetic risk models do not rely on self‐reporting and so are not at risk of recall or response bias. In the future, routine collection of genetic risk factors via a cheek swab or a pin‐prick blood sample may be easier than the collection of other data. Thirdly, genetic factors are largely independent of, and hence complimentary to, other risk factors [2]. Consequently, genetic risk models, unlike many phenotypic models, do not predispose towards older and sicker people [6]. There is also evidence, from a recent population‐based survey, that genetic risk models would be more acceptable to the general public than risk scores that use lifestyle risk factors, in the context of risk‐stratified screening for cancer [7].

The potential for genetic risk models to enhance disease risk prediction is appealing in the context of kidney cancer. A lack of symptoms, even at late stages of the disease, makes the detection of kidney cancer a challenge: 60% of kidney cancers in the UK are currently diagnosed incidentally and ~20% of those are late stage (III–IV) at diagnosis with associated poor 5‐year cancer‐specific survival rates (6% for Stage IV) [8]. Together with the observed increase in incidence of kidney cancer [9], this has led to international interest in the potential for a screening programme [10]. However, as the incidence of kidney cancer is relatively low in the general population [11], a targeted, risk‐stratified approach using risk models to identify high‐risk individuals most likely to benefit from screening is likely to be necessary [12, 13]. Risk models could also be used to guide choice of screening test and may provide opportunities for risk reduction interventions. In a previous validation study [14], we demonstrated that phenotypic risk models (incorporating lifestyle and demographic risk factors) that predict the development of kidney cancer have reasonable performance (95% CIs of the area under the receiver operating characteristic [AUROC] curve 0.50–0.71). However, the modelled incremental benefit over age was small. Adding genetic risk factors to phenotypic risk models has been shown to increase the discriminatory ability for other cancers [15].

In this review, we identify and evaluate existing models that both predict the development of kidney cancer and include genetic risk factors (either alone or in combination with other risk factors) to provide an overview of the current state of research in this area. We also assess the performance of the identified risk models in a large UK population (the UK Biobank [UKB] cohort) to enable a comparison between the included models and with genetic risk models for other cancers. A glossary of terms is provided in Boxes 1.

Box 1 **Glossary of Terms.**

SNP (single nucleotide polymorphism) – the most common type of genetic variation, SNPs refer to the difference of a nucleotide in a specific location in DNA (e.g., the replacement of the nucleotide cytosine [C] with the nucleotide thymine [T]).

GWAS (genome‐wide association studies) – a genome‐wide association study is an approach that involves scanning markers across complete sets of DNA of many individuals to find SNPs associated with a particular disease.

Discrimination (of a risk model) – a measure of how well a prediction model distinguishes between individuals with and without the outcome of interest. A model with discriminative ability will, on average, assign higher risk to the cases than the controls.

Calibration (of a risk model) – a measure of the agreement between the predicted and observed outcomes, the risk predicted by a model and observed risk.

AUROC (area under the receiver operating characteristic) curve – A ROC curve plots the sensitivity against 1‐specificity for a range of cut‐off points. The area under the curve is equal to the probability that an individual with the outcome is assigned a higher risk than a randomly chosen control. An AUROC value of 1.0 indicates a model with perfect discriminative ability, a value of 0.5 indicates discrimination no better than random assignment. Harrell’s concordance index (c‐index) is an equivalent measure used in open cohort (e.g., survival) analysis.

Population attributable fraction (PAF) – a widely used epidemiological measure of the fraction of all cases of a particular disease or other adverse condition in a population that is attributable to a specific exposure. This can be interpreted as the proportion of cases that would not have occurred if the exposure was not present.

Phenotypic – the observable characteristics of an individual resulting from the interaction of their genome with the environment. In this review, we refer to phenotypic models that may include demographic, lifestyle, and clinical risk factors.

PRS (polygenic risk score) – also referred to as genome‐wide score or genetic risk score summarise the estimated effect of many genetic variants (SNPs) on an individual. Here, we specifically use the term PRS to refer to models constructed from weights derived from a GWAS.

Cancer PRS web directory – an on‐line repository for polygenic risk scores for major cancer traits https://prsweb.sph.umich.edu:8443/

Germline mutations – mutations or variation association that are present in germ cells and can be passed on to offspring (as opposed to somatic mutations that occur outside of germline cells and cannot be passed on to offspring).

Truncating variants – a genetic variation that results in a shorter version of the associated protein being expressed, which can cause loss of function for the gene in which they are present.

Minor allele fraction (MAF) – the proportion at which the second most common allele occurs in a given population. Common variants are considered to be those with a MAF of >5% (although a cut‐off of >1% is not uncommon). Rare variants, while they can confer a high risk, will only be present in a small number of the cases and therefore will have little effect on the overall predictive accuracy of the model.

## Methods

We identified risk models from a recent systematic review [16] and the Cancer‐PRS web directory (an on‐line repository for PRSs for major cancer traits) [17]. We extracted data on the genetic risk factors (including how they were identified), the performance of the models in external validation studies and any comparisons to risk scores for other cancers.

The performance of the models was then assessed in the UKB cohort, a large population based cohort of ~500 000 individuals aged 40–69 years enrolled between 2006 and 2010 [18]. All participants attended a baseline assessment that included completion of questionnaires about lifestyle and medical history and measurement of a range of physical characteristics. Data on cancer incidence are available for UKB participants through linkage to national cancer registries. Full genotype information is available for 488 377 members of the UKB (Appendix S1). To maximise the number of cases, a closed‐cohort analysis with 6‐years of follow‐up was used for the validation. Cases of kidney cancer (all types) were included if they occurred within 6‐years of baseline assessment. Individuals with a diagnosis of kidney cancer prior to baseline (*n* = 452) were excluded from the analysis.

Two of the models included in this review, Fritsche et al. [17], uses SNPs that were originally identified as having an association with kidney cancer in a genome‐wide association study (GWAS) that used the UKB cohort. Therefore, the results presented for the Fritsche et al. [17] models cannot be considered true external validation. None of the other models used the UKB cohort as a development cohort or used SNPs identified in a GWAS that used the UKB cohort.

The performance, both discrimination and calibration, was measured for all of the models included in the validation. Discrimination was measured using the AUROC curve and the mean standardised score (MSS). Calibration was assessed graphically in deciles (Appendix S1). For models with sufficient unique values, we calculated the sensitivity, specificity, positive predictive value (PPV) and negative PV (NPV) for the deciles of the population with the highest and lowest scores.

A complete case approach was used for the primary analysis; each model was only computed for individuals with data for all of the risk factors used in that model. As this was done on a model‐by‐model basis, the cohort size varies slightly for each validation. Any phenotypic variables with >5% missing data were multiply imputed using a predictive mean matching approach (Appendix S1). Several sensitivity analyses were carried out; including stratified analyses to determine variation in performance by sex and ethnicity (Appendix S1).

## Results

A total of 22 studies describing 39 models that predict the risk of kidney cancer using genetic risk factors were identified and included in the narrative synthesis [15, 17, 19, 20, 21, 22, 23, 24, 25, 26, 27, 28, 29, 30, 31, 32, 33, 34, 35, 36, 37, 38].

### Genetic Risk Factors

In all, 90 genetic variants (SNPs) are used in the 39 models. The number of SNPs included in each of the models ranges from one (combined with other risk factors in a mixed genetic‐phenotypic models [21, 27, 28, 30]) to 19 [15, 26]. Details of the variables (including SNPs) used in each model are given in Table 1. Most of the SNPs (*n* = 63) are only used in a single study; however, the remainder of SNPs (*n* = 27) are implemented in models developed in more than one study. The most commonly used SNPs (rs2241261, rs11813268, rs10936602, rs74911261, rs4381241, rs718314) were used in models from six different studies. Further details of the SNPs used (including effect allele, minor allele fraction (MAF) and imputation score in the UKB) are given in Table S7.

**Table 1:** Included models.

Model ID	Number of SNPs	Other risk factors	Previous external validation (validation cohort)
Arjumand 2012	2	Smoking, age, sex, BMI, hypertension	
Arjumand 2012	2		
Chang 2014	2		
Chen 2011a	1	Smoking	
Chen 2011b	1	Smoking	
Chu 2012a	2		
Chu 2012b	2		
Chu 2012c	2		
Coric 2017	4		
DeMartino 2016	6	Smoking, age, sex, BMI, hypertension, **MNS16A (minisatellite tandem repeat)**	
Hsueh 2017a	1	**Urinary 8‐OHdG levels**	
Hsueh 2017b	1	**Urinary total arsenic**	
Hsueh 2018a	1	Smoking, age, sex, BMI, hypertension, education level, alcohol consumption, diabetes, **urinary creatinine levels, urinary total arsenic**	
Hsueh 2018b	1	Smoking, age, sex, BMI, hypertension, education level, alcohol consumption, **urinary creatinine levels, urinary total arsenic**	
Hsueh 2018c	1	Smoking, age, sex, BMI, hypertension, education level, alcohol consumption, diabetes, **urinary creatinine levels, urinary total arsenic**	
Hsueh 2018d	1	Age, sex, BMI, hypertension, education level, alcohol consumption, diabetes, **urinary creatinine levels, urinary total arsenic**	
Li 2012a	1	Smoking, age, sex, BMI, hypertension, education, ethnicity	
Li 2012b	1	Smoking, age, sex, BMI, hypertension, education, ethnicity	
Li 2012c	1	Smoking, age, sex, BMI, hypertension, education, ethnicity	
Lin 2008a	12	**XPC intron 9 (PAT)**	
Lin 2008b	7		
Machiela 2017a	9		
Machiela 2017b	9		
Scelo 2016	13		
Shu 2013	6		
Verma 2015	2		
Wei 2014a	5		
Wei 2014b	3		
Wu 2016a	3		
Wu 2016b	3		
Graff 2021	19		× (GERA, UKB)
Shi 2019a	10		× (TCGA, eMERGE)
Shi 2019b	10		× (TCGA, eMERGE)
Fritsche 2018	7		× (MGI)
Fritsche 2020a	12		× (MGI)
Fritsche 2020b	12		× (MGI, UKB)
Kachuri 2020	19		× (UKB)
Jia 2020	15		× (UKB)

Text in bold indicates variables not available for the UKB cohort.

GERA, Genetic Epidemiology Research on Aging; MGI, Michigan Genomics Initiative; 8‐OHdG, 8‐hydroxydeoxyguanosine; TCGA, The Cancer Genome Atlas.

Most of the SNPs included in the models were relatively common variants within the UKB cohort, with only seven rare alleles (MAF of <5%) identified. In particular, we note the models developed by Lin et al. [31], Fritsche et al. [25] and Fritsche et al. [17], which all used more than one rare allele (MAF of <%5) in their respective models.

Most of the included studies (*n* = 14), including all of those published before 2017, selected small numbers of SNPs of interest to include in models, based on known causal pathways for RCC [19, 20, 21, 22, 23, 24, 27, 28, 30, 31, 35, 36, 37, 38]. Variants on genes associated with vitamin D activity [19], immunoregulatory responses [22], susceptibility to stress [23], telomere length [24], DNA repair [28], adiponectin levels [27], the mammalian target of rapamycin (mTOR) pathway [35] and microRNA (miRNA) binding sites [36, 37] were all included by different studies based on hypothesised associations with kidney cancer. Additionally, genes with known associations to carcinogenesis [20], solid cancers [21], kidney cancer [30] and RCC [38] were selected by four of these studies.

Eight of the included studies, all published since 2017, used SNPs found to be associated with kidney cancer in GWAS [15, 17, 25, 26, 29, 32, 33, 34]. In GWAS, the whole genome of a large cohort is searched for association to the outcome of interest. This approach can identify large numbers of genetic variants, but biological mechanisms linking the identified SNPs to the outcome are not identified. Nine separate GWAS were given as sources for SNPs used in models included in this review (Table 4) [33, 39, 40, 41, 42, 43, 44, 45, 46]. Most (seven studies) used RCC as the outcome for which associations were identified [33, 39, 40, 41, 42, 43, 45], while one used the outcome of Wilms’ tumour [44] and one did not report the outcome [46]. The size of the GWAS populations ranged from 2636 [44] to 408 961 (the UKB cohort) [46], with the number of outcomes ranging from 757 [44] to 10 784 [33]. All of these GWAS except one [33], exclusively used White (often defined as European ancestry) populations.

### Genetic Risk Models

We identified 14 studies (describing 27 models) that used SNPs located in genes associated with known causal pathways for kidney cancer [19, 20, 21, 22, 23, 24, 27, 28, 30, 31, 35, 36, 37, 38]. All of these studies used a case–control design to develop models and most recruited patients with RCC as cases (Table S2). Furthermore, they all recruited majority male populations (57%–85%) with a mean age of >50 years. A range of ethnicities are represented, including Asian (eight ) [19, 20, 21, 22, 27, 28, 36, 38], White‐only (four) [23, 31, 35] and mixed ethnicity (one) [30]. The development populations range in size from 355 (100 cases, 225 controls) [36] to 2050 (894 cases, 1156 controls) [37]. The number SNPs included in these models ranges from one to 12 (Table 1). Only one study [38] reported the discrimination of any of these models in their development population, and to the knowledge of the authors there have been no prior external validations of these models. In all, 13 of these models included phenotypic risk factors alongside genetic factors [19, 21, 24, 27, 28, 30]. The most common included risk factors in the mixed models are smoking (10), sex (nine), body mass index (BMI; nine), age (eight), and hypertension (eight).

A further eight studies (12 models) combine SNPs identified through GWAS (eight studies and 12 models) [15, 17, 25, 26, 29, 32, 33, 34]. Both the SNPs and their weighting are determined in GWAS and then compiled to form a PRS. The number of SNPs used in these models ranges from seven to 19.

### Published Performance of Genetic Risk Models

Eight of the genetic‐only risk models included in this review have previously been validated in external populations [15, 17, 25, 26, 29, 34]. In most of these validations, the genetic model for kidney cancer is shown to have some ability to distinguish individuals at high risk (Table S3).

In the study by Kachuri et al. [15], the predictive value of adding a cancer‐specific PRS to a phenotypic model (including age, family history and modifiable lifestyle risk factors) is also evaluated. The discrimination, measured by the c‐index, for the kidney cancer model increased from 0.716 to 0.723 when adding the PRS to the model. The authors estimated that the population attributable fraction (PAF) for the genetic risk factors included in their model was 4.6%.

All of the external validation studies used populations from the UK and USA and all limited to either European ancestry [15, 17, 25, 26, 29], Caucasians [34] or self‐reported White individuals [17]. Additionally, all use kidney cancer (all types, excluding renal cancer of the pelvis), not RCC, as the outcome of interest.

### Comparable Performance of Genetic Risk Models

We validated 31 of the identified models in the UKB cohort [15, 17, 20, 21, 22, 23, 24, 25, 26, 29, 30, 31, 32, 33, 34, 35, 36, 37, 38]. Eight models were not validated either because some of the variables included were not available for the UKB cohort [27, 28] or because the information required to validate the models was not available [19].

**Fig. 1:**
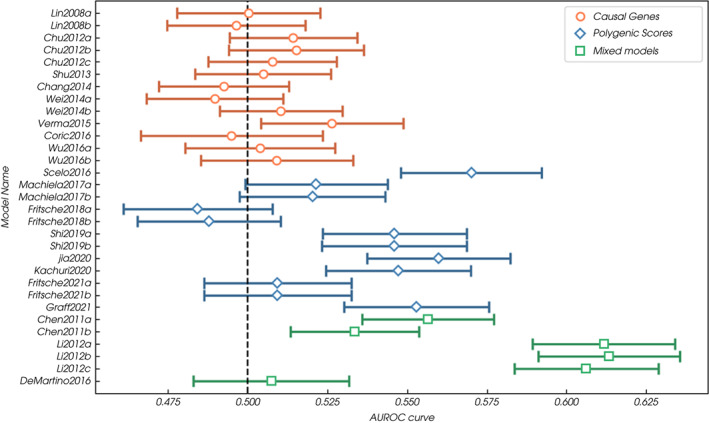
Discrimination (AUROC curve) of all models incorporating genetic risk factors included in the validation analysis. [Colour figure can be viewed at wileyonlinelibrary.com]

**Fig. 2:**
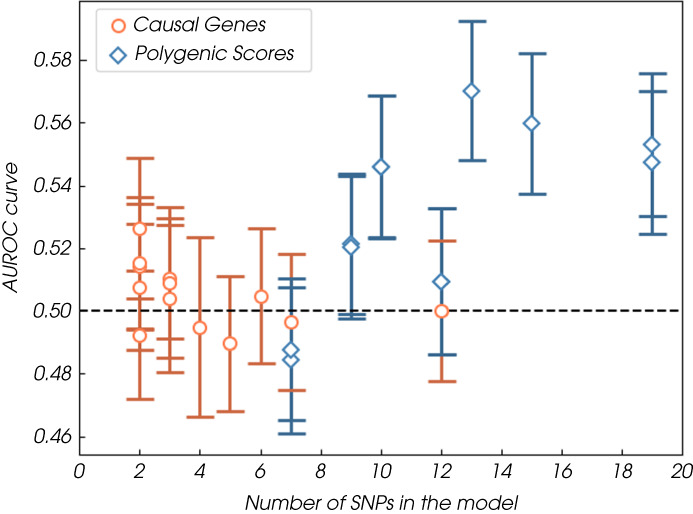
Discrimination (AUROC curve) of all of all models incorporating genetic risk factors included in the validation analysis plotted by number of SNPs included in the model. [Colour figure can be viewed at wileyonlinelibrary.com]

We included 438 315 individuals from the UKB cohort, including 620 cases of kidney cancer, in the primary analysis (Table 2). In this cohort, the six genetic‐only models with the highest discrimination (all with adequate calibration) used SNPs derived from GWAS [15, 26, 29, 33, 34] (Fig. 1). Of these, the PRS by Scelo et al. [29] had the highest discrimination (AUROC curve 0.551, 95% CI 0.528–0.573). This model also has the highest odds ratio (OR) per standard deviation (SD) of risk score, 1.189 (SE 0.051). The Scelo et al. [29] model is adequately calibrated; with some overestimation in the high‐risk deciles (see Appendix S1 for plots). The genetic‐only models with the highest sensitivity (14.3%) and PPV (0.20%) for the 10% of the population with the highest scores are the two developed by Shi et al. [34], which use 10 SNPs weighted for the development and validation populations respectively (Table S5). The model developed by Jia et al. [29], which includes 15 SNPs, has the lowest sensitivity (6.7%) and PPV (0.094%) for the 10% of the population with the lowest scores. Of the genetic‐only models using variants inferred from a causal pathway, only the model developed by Verma et al. [36], which used SNPs from miRNA genes previously shown to be associated with solid cancers, had discriminative ability (AUROC curve 0.526, 95% CI 0.504–0.549); however, calibration is poor. No other genetic‐only models showed discriminative ability (Table 2, Fig. 1). In general, the discrimination of the genetic‐only models improves as more SNPs are added to the models (Fig. 2).

**Table 2:** UKB cohort characteristics.

		All	Controls	Cases	*P* ^*^	% Incident KCa
Counts	*n*	435 572	434 957	615		0.1412
Age, years	Mean (sd)	56.5 (8.09)	56.5 (8.09)	61.0 (6.19)	<0.001	–
	Missing, *n* (%)	0 (0)	0 (0)	0 (0)		–
Sex	Female, *n* (%)	236 149 (54.2)	235 927 (54.2)	222 (36.1)	–	0.0940
	Male, *n* (%)	199 423 (45.8)	199 030 (45.7)	393 (63.9)	<0.001	0.1971
	Missing, *n* (%)	0 (0)	0 (0)	0 (0)		–
Ethnicity	White, *n* (%)	413 002 (94.8)	412 398 (94.8)	604 (98.2)	0.024	0.1462
	Mixed heritage, *n* (%)	2408 (0.55)	2407 (0.55)	1 (0.16)		0.0415
	South East Asian, *n* (%)	7805 (1.79)	7800 (1.79)	5 (0.81)		0.0641
	Black, *n* (%)	6022 (1.38)	6019 (1.38)	3 (0.49)		0.0498
	Chinese, *n* (%)	1282 (0.29)	1282 (0.29)	0 (0)		0.0000
	Other, *n* (%)	3616 (0.83)	3614 (0.83)	2 (0.33)		0.0553
	Missing, *n* (%)	1437 (0.33)	1437 (0.33)	0 (0)		0.0000
BMI, kg/m^2^	Median (IQR)	26.7 (24.1–30.0)	26.7 (24.1–30.0)	28.0 (25.4–31.5)	<0.001	–
	<20, *n* (%)	10 108 (2.32)	10 099 (2.32)	9 (1.46)		0.0890
	20–24.9, *n* (%)	133 479 (30.6)	133 364 (30.7)	115 (18.7)		0.0862
	25–29.9, *n* (%)	184 866 (42.4)	184 586 (42.4)	280 (45.5)		0.1515
	≥30, *n* (%)	105 546 (24.2)	105 337 (24.2)	209 (34.0)		0.1980
	Missing (%)	1573 (0.36)	1571 (0.36)	2 (0.33)		0.1271
Smoking status	Never, *n* (%)	238 042 (54.7)	237 776 (54.7)	266 (43.3)	<0.001	0.1117
	Former, *n* (%)	151 068 (34.7)	150 803 (34.7)	265 (43.1)		0.1754
	Current, *n* (%)	46 462 (10.7)	46 378 (10.7)	84 (13.7)		0.1808
	Missing, *n* (%)	2503 (0.56)	2498 (0.56)	5 (0.63)		0.1998
Blood pressure (BP), mmHg	Systolic BP, mean (sd)	138.1 (18.7)	138.0 (18.7)	145.2 (19.5)	<0.001	–
	Diastolic BP, mean (sd)	82.3 (10.2)	82.3 (10.2)	84.1 (10.7)	<0.001	–
Alcohol consumption^†^	Never, *n* (%)	34 612 (8.0)	34 568 (8.0)	44 (7.1)	0.720	0.1273
	Light drinkers, *n* (%)	213 361 (49.0)	213 053 (49.0)	308 (50.1)		0.1446
	Heavy drinkers, *n* (%)	187 215 (43.0)	186 953 (43.0)	262 (42.6)		0.1401
	Missing, *n* (%)	384 (0.09)	383 (0.09)	1 (0.16)		0.2611
Previous cancer diagnosis, *n*	0, *n* (%)	395 849 (90.9)	395 314 (90.9)	535 (87.0)	<0.001	0.1352
	1, *n* (%)	29 125 (6.7)	29 068 (6.7)	57 (9.3)		0.1957
	≥2, *n* (%)	10 598 (2.4)	10 575 (2.4)	23 (3.7)		0.2170

IQR, interquartile range; KCa, kidney cancer.

^*^ Tested for difference in means using *t*‐test, difference in medians using Wilcoxon rank‐sum test and difference in frequency of cases between categories with Pearson’s chi‐squared test.

^†^ Light drinkers report <14 units/week, heavy drinkers report ≥14 units/week.

Five of the six mixed phenotypic‐genetic models included in the validation showed discriminative ability (lower bound of the AUROC curve >0.5) [21, 30] (Table 3, Fig. 1). Of these, the best performing are the three models developed by Li et al. [30], which all combine a single SNP (from the apolipoprotein E [APOE] promoter region) with seven phenotypic risk factors, including age and smoking (95% CI range of the AUROC curve 0.584–0.636, calibration adequate, underestimation by the model in high‐risk deciles).

**Table 3:** Primary analysis (external validation in the UKB): discrimination measures (models ordered by type and publication date).

Model name	Type	Cases, *n*	Cohort, *n*	AUROC curve (95% CI)
Lin 2008a	Causal genes	615	435 572	0.500 (0.478–0.523)
Lin 2008b	Causal genes	605	426 092	0.496 (0.475–0.518)
Chu 2012b	Causal genes	610	433 036	0.515 (0.494–0.536)
Chu 2012a	Causal genes	610	431 465	0.514 (0.494–0.534)
Chu 2012c	Causal genes	612	432 797	0.508 (0.487–0.528)
Shu 2013	Causal genes	615	435 572	0.505 (0.483–0.526)
Wei 2014b	Causal genes	593	415 664	0.51 (0.491–0.530)
Chang 2014	Causal genes	613	433 109	0.492 (0.472–0.513)
Wei 2014a	Causal genes	615	435 572	0.49 (0.468–0.511)
Verma 2015	Causal genes	604	428 885	0.526 (0.504–0.549)
Wu 2016b	Causal genes	615	435 572	0.509 (0.485–0.533)
Wu 2016a	Causal genes	615	435 572	0.504 (0.48–0.527)
Coric 2016	Causal genes	615	435 572	0.492 (0.471–0.513)
Scelo 2017	PRS	615	435 572	0.551 (0.528–0.573)
Machiela 2017a	PRS	615	435 572	0.521 (0.499–0.544)
Machiela 2017b	PRS	615	435 572	0.520 (0.497–0.543)
Fritsche 2018b	PRS	614	434 969	0.488 (0.465–0.510)
Fritsche 2018a	PRS	614	434 969	0.484 (0.461–0.508)
Shi 2019a	PRS	615	435 572	0.546 (0.523–0.569)
Shi 2019b	PRS	615	435 572	0.546 (0.523–0.569)
Jia 2020	PRS	615	435 572	0.560 (0.537–0.582)
Kachuri 2020	PRS	615	435 572	0.547 (0.525–0.57)
Graff 2021	PRS	615	435 572	0.553 (0.530–0.576)
Fritsche 2021a^*^	PRS	614	434 969	0.509 (0.486–0.533)
Fritsche 2021b^*^	PRS	614	434 969	0.509 (0.486–0.533)
Chen 2011a	Mixed	612	434 120	0.556 (0.536–0.577)
Chen 2011b	Mixed	612	434 439	0.534 (0.513–0.554)
Li 2012b	Mixed	608	425 903	0.614 (0.592–0.636)
Li 2012a	Mixed	608	425 903	0.612 (0.59–0.635)
Li 2012c	Mixed	608	425 903	0.607 (0.584–0.629)
DeMartino 2016	Mixed	516	368 911	0.506 (0.481–0.53)

^*^ Some of the SNPs identified in a GWAS study using UKB participants, so not a true external validation.

The supplementary analyses revealed no clear difference in discrimination between men and women or between the entire cohort and White‐only members of the UKB. When removing one of each set of third‐degree relatives from the cohort, the six highest performing genetic‐only models [15, 26, 29, 33, 34] had no significant differences in discrimination (95% CI of the AUROC curves 0.510–0.571), suggesting that in unrelated individuals in these six models would be expected to have similar performance. The results from all sensitivity analyses can be found in Table S8.

Note that at least two of the models validated in this study, developed by Fritsche et al. [17], use SNPs identified in GWAS of the UKB cohort (Table 4). There have also been previously reported external validations of several of the models that have used the UKB cohort (Table S3) [15, 17, 26, 29]. The results of this study are in agreement with these previous validations.

**Table 4:** GWAS studies used to develop PRSs.

Study (first author, year)	Studies that used SNPs identified in this GWAS	Disease	Country	Ethnicity	Cases	Controls	Named cohorts
Scelo 2017	Jia 2020 Kachuri 2020 Fritsche 2020 Shi 2019 Graff 2021 Scelo 2017	RCC	Norway, Slovakia, France, UK, Russia, Finland, and USA (plus other European countries)	A mixture of White and cohorts of unrestricted ethnicity	10 784	20 406	International Agency for Research on Cancer (IARC) MD Anderson Cancer Patients (MDA) Mayo Clinic Cohort
Henrion 2015	Jia 2020 Kachuri 2020 Shi 2019 Scelo 2017	RCC	UK, various European countries, and USA	White/European ancestry	2498	8799	UK‐GWAS (MRC SORCE and ICR/RM NHS, WTCCC2 UK Blood Service) US National Cancer Institute (NCI)
Purdue 2011	Jia 2020 Shi 2019 Scelo 2017	RCC	Various European countries, USA, UK	European background	3772	8505	IARC Centre National de Gènotypage (CNG) NCI
Henrion 2012	Jia 2020 Shi 2019 Scelo 2017	RCC	UK	White/European ancestry	1045	5200	UK‐GWAS (MRC SORCE and ICR/RM NHS, WTCCC2 UK Blood Service)
Wu 2012	Jia 2020 Shi 2019 Scelo 2017	RCC	USA	European descent (self‐reported Caucasian)	894	1516	MDA
Zhou 2018	Fritsche 2020	Unclear	UK	White British	408 961		UKB
Turnbull 2012	Fritsche 2020 Fritsche 2018	Wilms’ tumour	UK, USA	European ancestry	757	1879	Factors Associated with Childhood Tumours (FACT) Children’s Cancer and Leukaemia Group (CCLG) National Wilms’ Tumour Study Group (NWTSG) Children’s Oncology Group (COG)
Han 2012	Jia 2020	RCC	USA, Finland, Russia, Romania, Poland, and Czech Republic	European background	2278	3719	Prostate, Lung, Colorectal and Ovarian Cancer Screening Trial (PLCO) Alpha‐Tocopherol, Beta‐Carotene Cancer Prevention (ATBC) Central and Eastern European RCC (CEERCC) US Kidney Cancer (USKC)
Gudmundsson 2013	Jia 2020	RCC	Iceland, the Netherlands	Self‐reported European descent	2411	71 497	Icelandic RCC sample collection deCODE genetics Dutch RCC sample series Comprehensive Cancer Centre East

### Comparable Performance of Kidney Genetic Risk Models to Genetic Risk Models for Other Cancers

Several of the included validation studies reported the performance of kidney cancer risk models in comparison to risk models for other cancers in the same cohort. Compared to the best performing genetic‐only models for other types of cancer, the performance of the kidney cancer genetic models is relatively poor. In four of the six identified validation studies [15, 17, 25, 29], the kidney cancer model has the lowest or second lowest performance of all the cancer‐specific genetic risk scores evaluated. In most of these validations, the kidney cancer model is outperformed by models for more common cancers with a greater number of associated SNPs (including breast, prostate and colorectal, but not lung). For example, in a study by Jia et al. [29], they report that individuals with the highest 5% (cancer‐specific) PRS have a two–three‐times elevated risk of cancer of the prostate, breast, pancreas, colorectal and ovary, but only a 1.5‐times elevated risk of lung, bladder or kidney cancer. In their validation, the genetic risk model (included in this review) for kidney cancer had the lowest AUROC curve value of the eight cancer types examined. In the validation of genetic risk models for 16 types of cancer by Kachuri et al. [15], the increase in discrimination observed when adding a genetic risk score (included in this review) to models with other risk factors for kidney cancer (c‐index 0.716–0.723) is also the second lowest of the included cancer types. The increase in discrimination is much lower than for breast cancer (where the c‐index increased from 0.572 to 0.635) but comparable to that seen for bladder cancer (where the c‐index increased from 0.808 to 0.814). The PAF for the genetic risk included in the kidney cancer model (4.6%) is also lower than seen for bladder cancer (8.5%) or colorectal cancer (16.8%). However, in other validations the kidney cancer model performs adequately compared to genetic models for other cancers. In Graff et al. [26], the kidney cancer model (included in this review) ranks 11th out of 15 evaluated, with an effect size per SD (OR 1.21, 95% CI 1.14–1.26) higher than four other scores, including the PRS for oral cancer (OR 1.08, 95% CI 1.02–1.14) and the PRS for lung cancer (OR 1.12, 95% CI 1.08–1.17).

## Discussion

In this review, we have identified all existing models that use genetic risk factors that predict the risk of developing kidney cancer, and then validated the majority in the UKB cohort. At least 39 risk models incorporating 90 different genetic variants have been developed to predict the risk of kidney cancer. Several genetic‐only risk models demonstrate potential to discriminate between those at higher and lower risk of kidney cancer (lower bound of the AUROC curve >0.5). However, the best performing genetic‐only model has an AUROC curve value of 0.551 (95% CI 0.528–0.573) [33], considerably lower than the AUROC curve seen for genetic‐only risk models in some other cancers. The incremental benefit of adding a genetic risk model for kidney cancer to a phenotypic risk model is also marginal (an increase in the AUROC curve of 0.007 from 0.716 [SE 0.011] to 0.723 [SE 0.011]), and lower than observed for other cancers (the AUROC curve increases by 0.063 from 0.572 [SE 0.005] to 0.635 [SE 0.004] for breast cancer) [15].

The performance of the kidney cancer models in the UKB also compares poorly with genetic risk models for other cancers validated in the UKB. For example, the genetic model developed by Huyghe et al. [47] for colorectal cancer has a AUROC curve value of 0.63 (95% CI 0.61–0.64) [48] and the model developed by Mavaddat et al. [49] for breast cancer has an AUROC curve value of 0.63 (95% CI 0.63–0.65) in a validation cohort of women (largely drawn from the UKB).

Two observations suggest that the comparatively poor performance of current genetic risk models for predicting kidney cancer is probably due to the limited number of SNPs currently identified and included within the models. Firstly, the number of SNPs included in the kidney cancer models is considerably lower than for other cancers. In Graff et al. [26], 19 SNPs are included in the kidney cancer model (the highest number of any model included in this review), whereas in the same study 103 and 187 SNPs are used in the scores for colorectal and breast cancer, respectively. Further, the analysis in this review suggests that discrimination improves as the number of SNPs increases (Fig. 2). This has been seen in other cancers, e.g., in a previous validation of genetic risk models for colorectal cancer (also in the UKB) models with similar numbers of SNPs; Yarnall 2013 (15 SNPs) [50] and Ibanez‐Sanz 2017 (23 SNPs) [51] have comparable performance (AUROC curves of 0.56, 95% CI 0.54–0.57; and 0.56, 95% CI: 0.54–0.58) to the Graff et al. [26] model (19 SNPs) for kidney cancer. The best performing model [47] from that validation of includes 120 SNPs and has an AUROC curve of 0.63 (95% CI 0.61–0.64).

Secondly, the PAF for one of the best genetic‐only risk models for kidney cancer included in this review [15] (19 SNPs) is estimated to be only 4.6%. However, a study of environmental and heritable risk, using a large Nordic cohort of twins, estimates that the true PAF of genetic risk factors for kidney cancer could be as high as 38% [52]. Similarly, a 2015 study found that that the genetic variants identified by GWAS (at that time) explained only 14.7% of the heritability associated with kidney cancer [53]. This suggests that there may be up to 100 SNPs associated with kidney cancer risk that have not yet been identified.

The limited number of SNPs identified to date is likely due to the relatively small number of GWAS for kidney cancer. Compared with the nine GWAS studies used to develop kidney cancer risk models [33, 39, 40, 41, 42, 43, 44, 45, 46], there have been >100 different breast cancer GWAS [54]. If the potential for genetic risk models for kidney cancer is going to be realised, there is a need for further GWAS studies to identify as of yet unknown variants associated with the development of this disease. Given the relatively low prevalence of kidney cancer (0.17, 95% CI 0.09–0.27, in Europe [11]), larger cohort sizes or longer follow‐up periods than studies for more common cancers will likely be needed to include sufficient case numbers in the analysis.

Alongside these efforts to identify further SNPs, there are also a number of other areas that need considering before any of these genetic risk models can be incorporated into clinical practice. Perhaps the most significant is the lack of data from individuals of non‐White ethnicity. Given the small numbers of individuals who self‐report non‐White ethnicity in the UKB, it was not possible to conduct analyses stratified by ethnicity in the validation performed in this review. The best performing genetic models use SNPs identified in GWAS that included almost exclusively White‐only populations (Table S3) and all previous external validations have excluded all non‐White individuals from their analyses (Table S2). The performance of these models across different ethnic groups is, therefore, a key question for this area of research. This is not unique to kidney cancer, a lack of ethnically diverse populations is a challenge across the field of genetics [55], with nearly 80% of individuals included in published GWAS being of European descent [56]. There is an urgent need for the prioritisation of genetic data generation from individuals from under‐represented ethnic groups (including African and Asian ancestries) [2]. Other considerations common across all cancers include how best to collect, store, and share genetic data [57]; how to communicate the results of genetic risk scores to individuals to minimise any psychosocial harms; how to address the training needs of healthcare professionals; and the need for clear regulatory frameworks to ensure responsible and equitable use of genetic risk models [2]. Modelling and cost‐effectiveness analyses are also needed to assess the potential benefits of incorporating genetic‐risk based stratification within the specific context of potential kidney cancer screening programmes once a suitable model had been developed.

Although it is encouraging to see the potential for genetic risk models to predict the development of kidney cancer, their relatively weak performance leads us to conclude that this area of research is not yet ready for transition into clinical practice. The low discrimination of even the best models included in this validation, means that they would not be as good as existing phenotypic models at selecting high‐risk individuals for screening. Although there has been rather limited research into combining genetic and phenotypic models for kidney cancer, the recent study showing that the Kachuri et al. [15] genetic model only marginally improved the performance of a phenotypic model is not promising. Without compelling evidence that the use of a genetic model could lead to a significantly better selection of high‐risk individuals, the additional expense and burden of collecting genetic information cannot be justified.

## Conclusions

While 90 genetic risk factors have been included in nearly 40 published genetic models predicting the risk of the development of kidney cancer, only a small number of these show any discriminative ability and the addition of genetic risk to phenotypic risk models results in only marginal improvement [15].

Overall, the best genetic models for kidney cancer perform poorly compared to the best genetic models developed for other cancers. Estimates suggest that the currently identified SNPs account for only 10%–20% of hereditable risk for kidney cancer. This may be due to the relatively small number of GWAS studies carried out for kidney cancer outcomes compared with those for other cancers, and hence, the relatively small number of variants associated with kidney cancer that have been identified.

Therefore, although in principle it is possible to identify individuals at higher risk of kidney cancer using existing models, these models are unlikely to have utility within clinical practice. If more, large GWAS studies are conducted, and more variants associated with kidney cancer are identified it seems likely that the development of higher performing PRSs will be achievable. Whether these will have utility within future kidney cancer screening pathways is yet to determined. On‐going research in other disease areas is also needed to ensure the responsible and equitable use of genetic risk scores in this context [2].

## Disclosures of Interest

Grant D. Stewart has received educational grants from Pfizer, AstraZeneca and Intuitive Surgical; consultancy fees from Pfizer, Merck, EUSA Pharma and CMR Surgical; Travel expenses from Pfizer, and Speaker fees from Pfizer. All other authors have no financial disclosures.

## Funding

Hannah Harrison was supported by a National Institute of Health Research Development and Skills Enhancement Award (NIHR301182) and is now supported by an International Alliance for Cancer Early Detection Project Award (ACEDFR3_0620I135PR007). Sabrina H. Rossi is supported by The Urology Foundation and a Cancer Research UK Clinical Research Fellowship. Grant D. Stewart’s work on this topic is funded by Kidney Cancer UK, The Urology Foundation, The Rosetrees Trust, Yorkshire Cancer Research and Cancer Research UK and supported by The Mark Foundation for Cancer Research, the Cancer Research UK Cambridge Centre [C9685/A25177] and NIHR Cambridge BRC. The University of Cambridge has received salary support in respect of Simon J. Griffin from the NHS in the East of England through the Clinical Academic Reserve. Juliet A. Usher‐Smith was funded by a Cancer Research UK Prevention Fellowship (C55650/A21464) and is now supported by a National Institute of Health Research Advanced Fellowship (NIHR300861). The views expressed are those of the author(s) and not necessarily those of the NIHR or the Department of Health and Social Care.

## Ethical Approval

The UKB study was approved by the North West Multi‐Centre Research Ethics Committee (reference number 06/MRE09/65), and at recruitment all participants gave informed consent.

## Informed Consent

Informed written consent to participate in UKB and be followed up, using a signature capture device.

**Abbreviations**
AUROC: area under receiver operating characteristic (curve)
BMI: body mass index
GWAS: genome‐wide association studies
MAF: minor allele fraction
miRNA: microRNA
(N)(P)PV: (negative) (positive) predictive value
OR: odds ratio
PAF: population attributable fraction
PRS: polygenic risk score
SNP: single nucleotide polymorphism
UKB: UK Biobank

## Supporting information


**Fig. S1** **.** (**a**) Sensitivity analysis comparing the model discrimination (AUROC curve) in men and women. (**b**) Sensitivity analysis comparing the model discrimination (AUROC curve) in the whole cohort and the White‐only cohort. (**c**) Sensitivity analysis comparing the model discrimination (AUROC curve) in the whole cohort and cohorts excluding individuals with multiple close relatives and individuals with any third degree relatives.

**Fig. S2** **.** (**a**) Calibration plots for GWAS models. (**b**) Calibration plots for causal gene models. (**c**) Calibration plots for mixed genetic and phenotypic models.

**Fig. S3** **.** Selection process of UKB cohort for primary analysis.

**Table S1** **.** Details of included models.

**Table S2** **.** Included studies and their development populations.

**Table S3** **.** Previously published external validations.

**Table S4** **.** (a and b) Use of UKB phenotypic variables.

**Table S5** **.** Primary analyses (external validation in the UKB): model accuracy in deciles.

**Table S6** **.** Primary analyses (external validation in the UKB): alternative measures of discrimination.

**Table S7** **.** Details of the single nucleotide polymorphisms (SNPs) used in the analysis.

**Table S8** **.** Model discrimination (AUROC) in sensitivity analyses.

**Appendix S1** **.** Supplementary Methods Section.
