# Supplementary material for: The current state of genetic risk models for the development of kidney cancer: a review and validation
Source: BJU Int. 2022 May 7;130(5):550–61. doi: 10.1111/bju.15752 (PMC9790357; doi:10.1111/bju.15752)
Supplement: Supplementary file 1 — Fig. S1 . (a) Sensitivity analysis comparing the model discrimination (AUROC curve) in men and women. (b) Sensitivity analysis comparing the model discrimination (AUROC curve) in the whole cohort and the White‐only cohort. (c) Sensitivity analysis comparing the model discrimination (AUROC curve) in the whole cohort and cohorts excluding individuals with multiple close relatives and individuals with any third degree relatives. [file BJU-130-550-s003.pptx]

## Slide 1
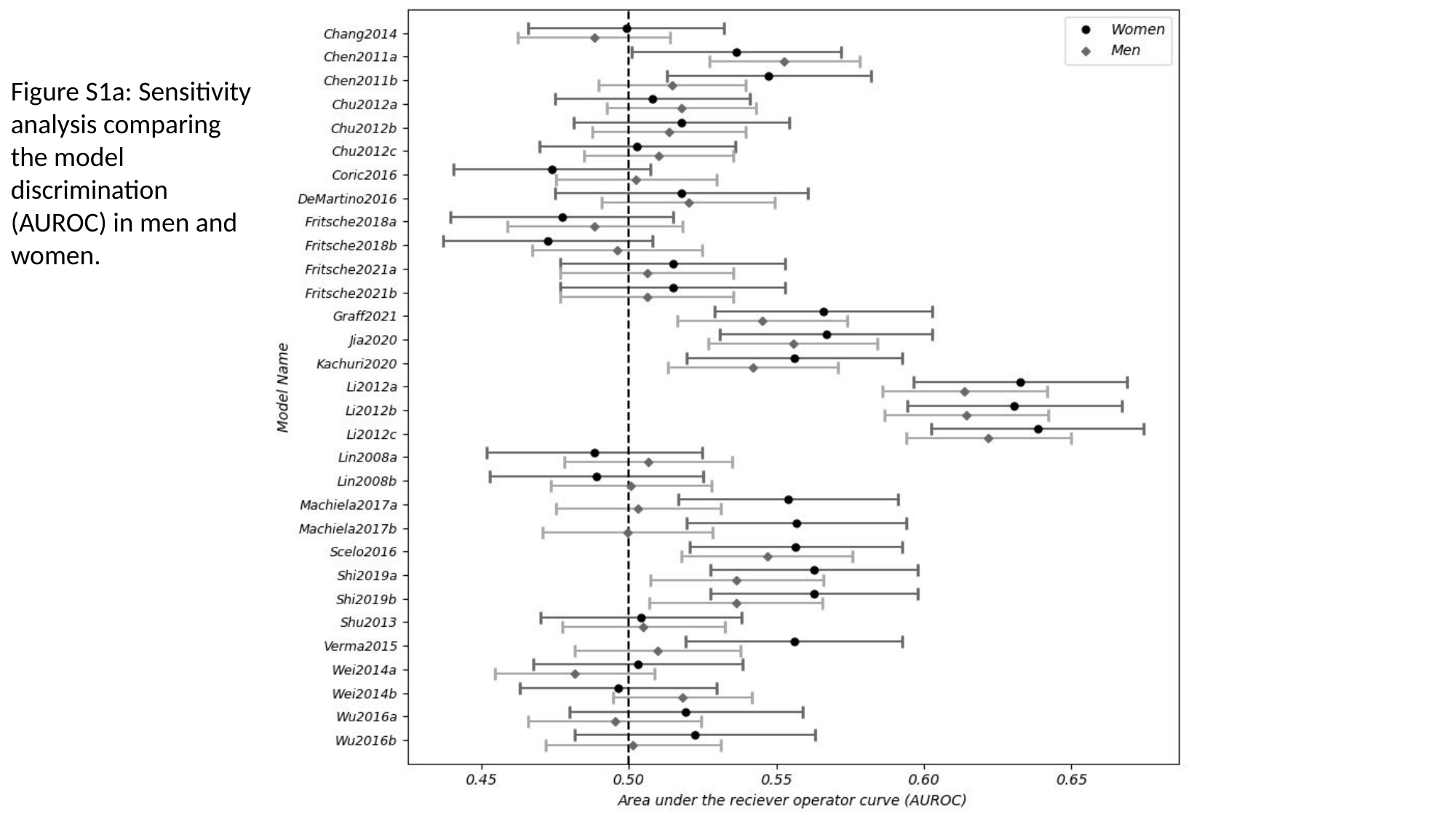

Figure S1a: Sensitivity analysis comparing the model discrimination (AUROC) in men and women.

## Slide 2
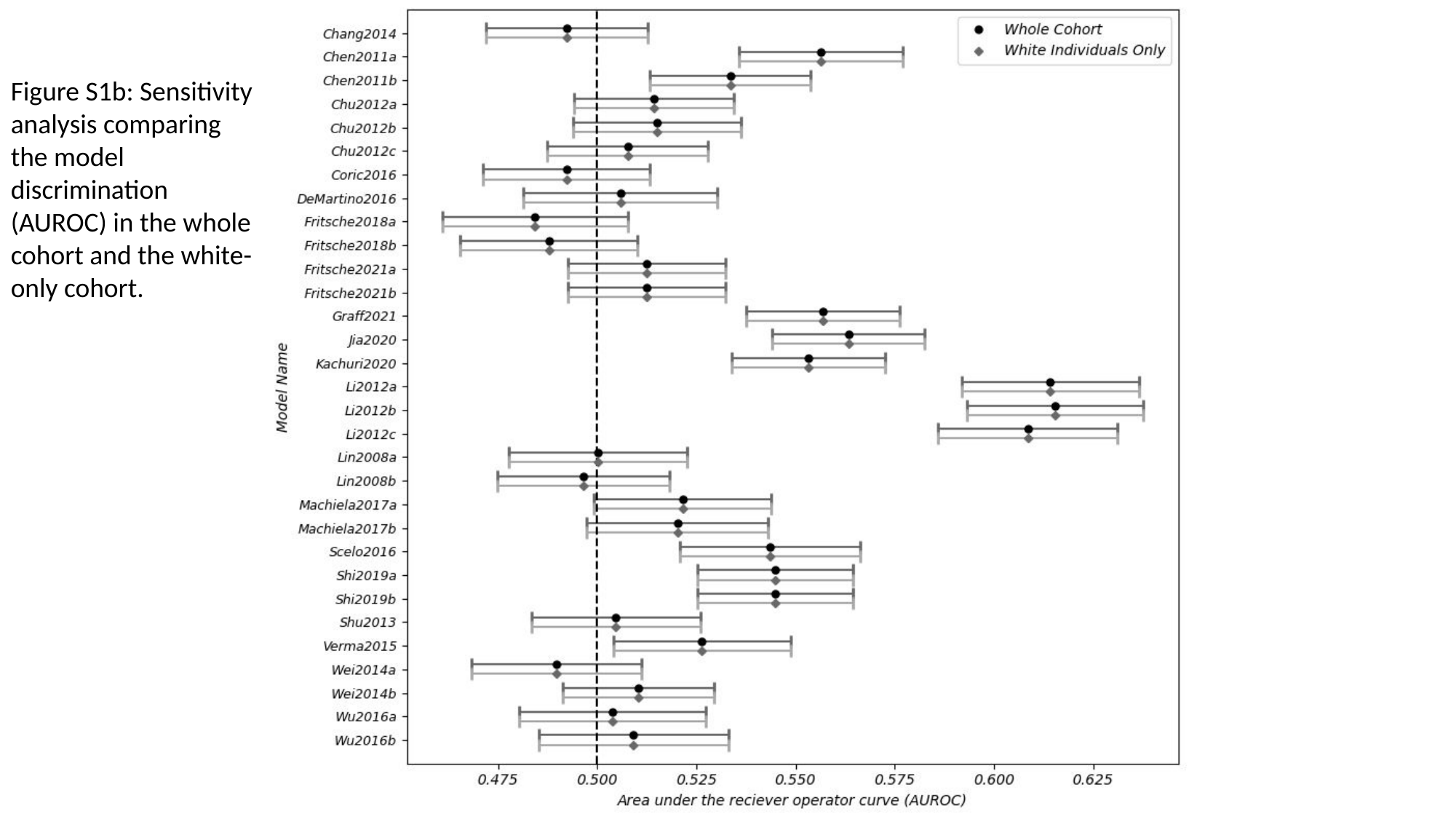

Figure S1b: Sensitivity analysis comparing the model discrimination (AUROC) in the whole cohort and the white-only cohort.

## Slide 3
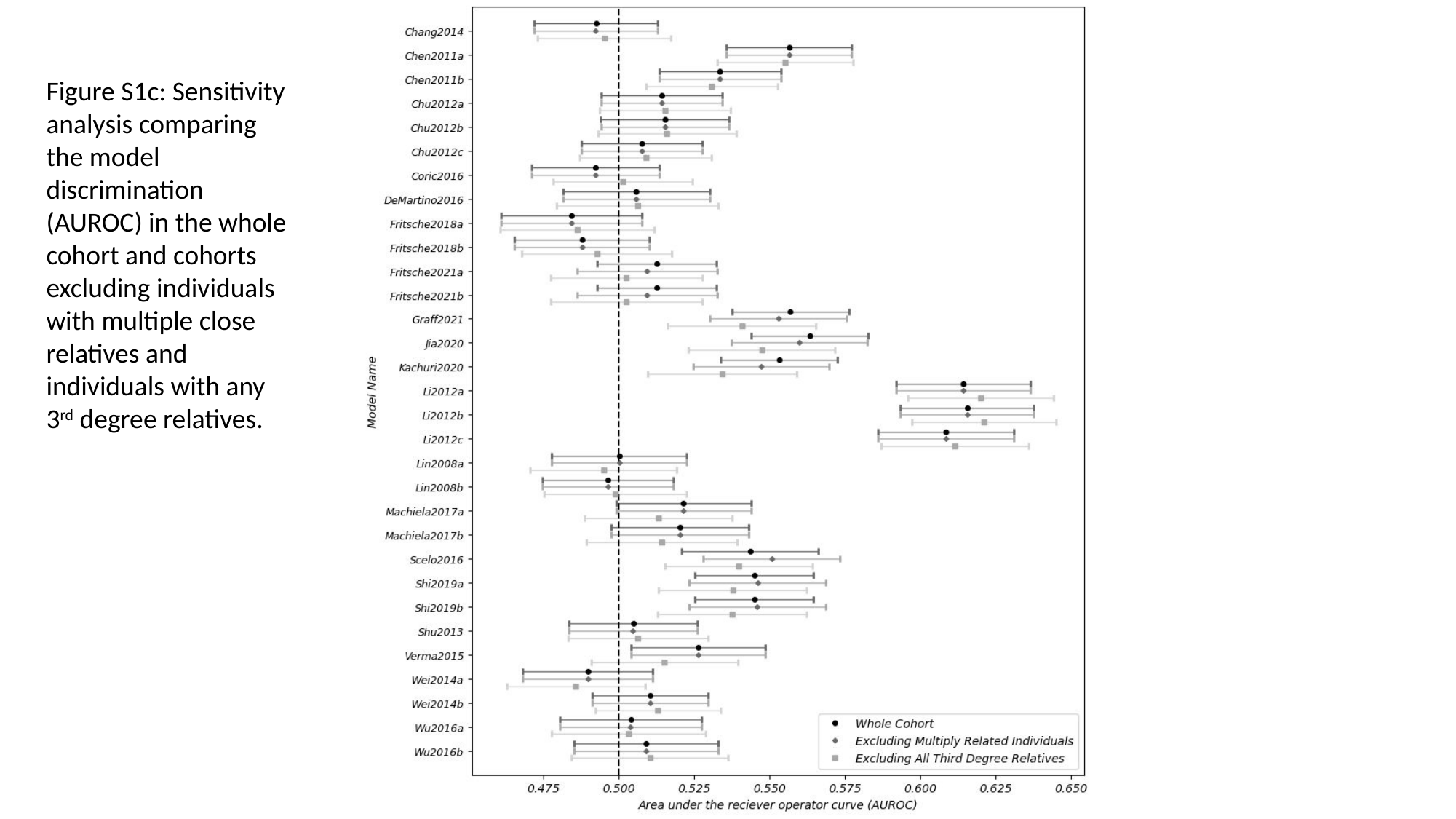

Figure S1c: Sensitivity analysis comparing the model discrimination (AUROC) in the whole cohort and cohorts excluding individuals with multiple close relatives and individuals with any 3rd degree relatives.

## Slide 4
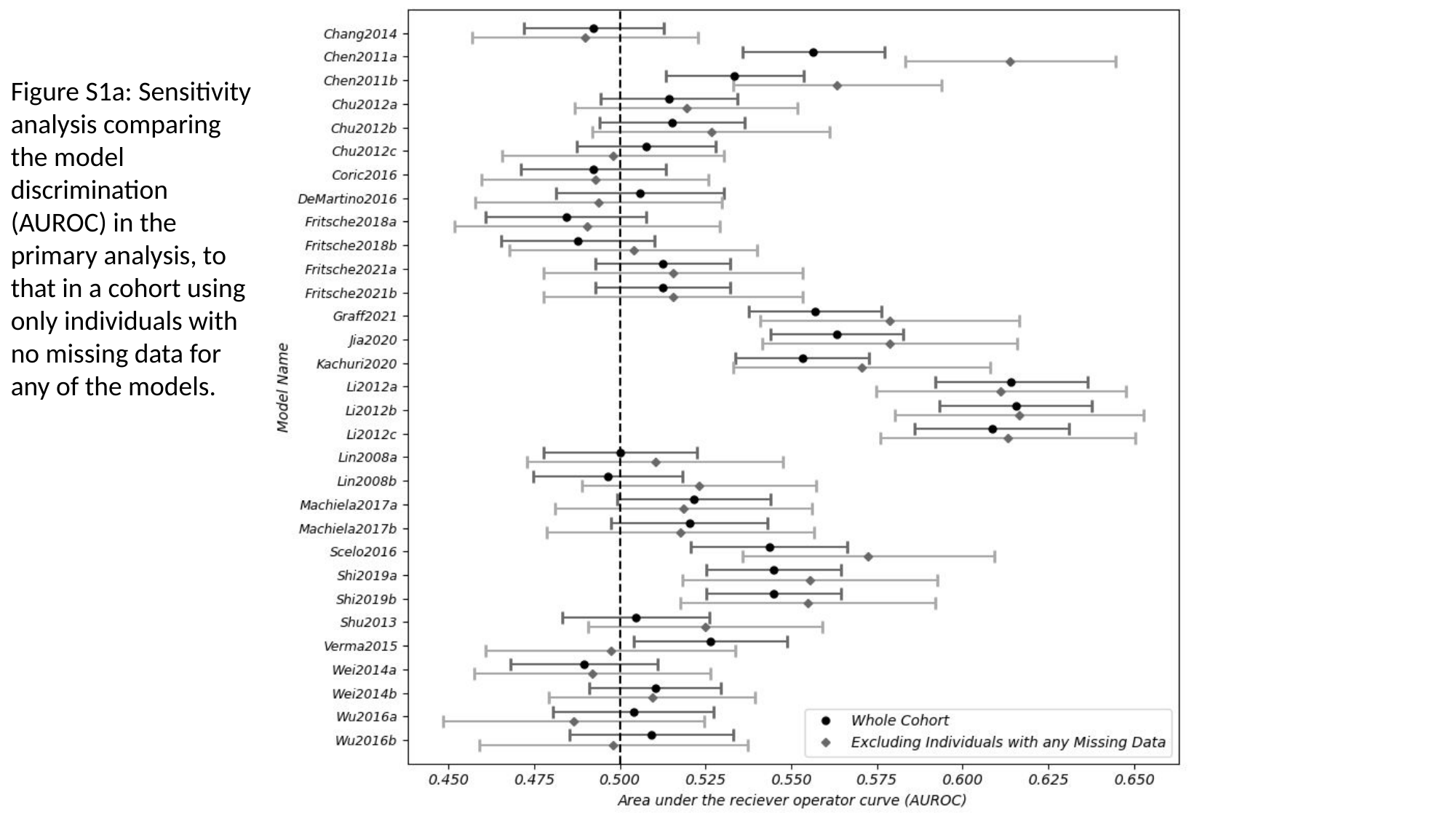

Figure S1a: Sensitivity analysis comparing the model discrimination (AUROC) in the primary analysis, to that in a cohort using only individuals with no missing data for any of the models.

## Slide 5
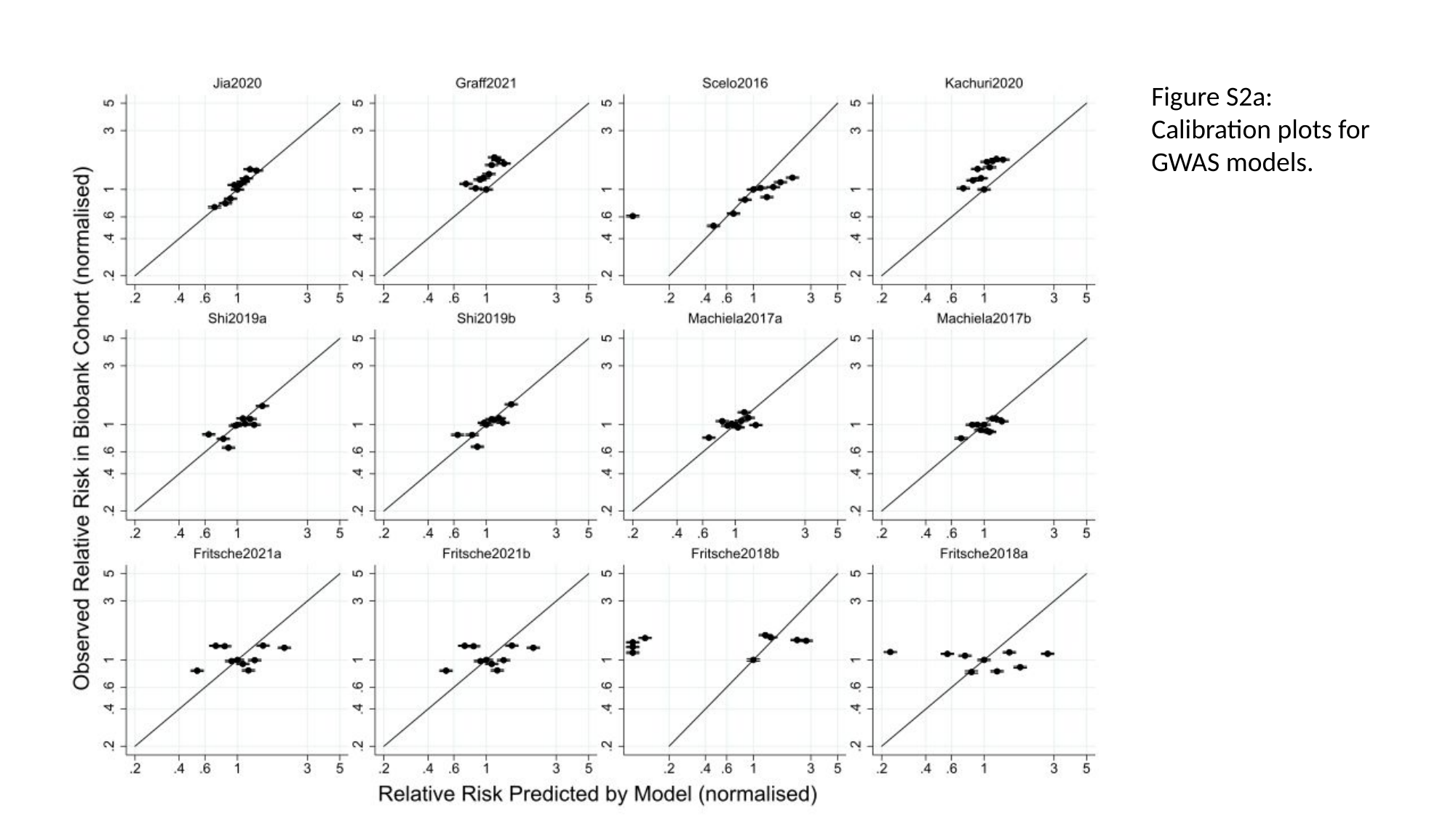

Figure S2a: Calibration plots for GWAS models.

## Slide 6
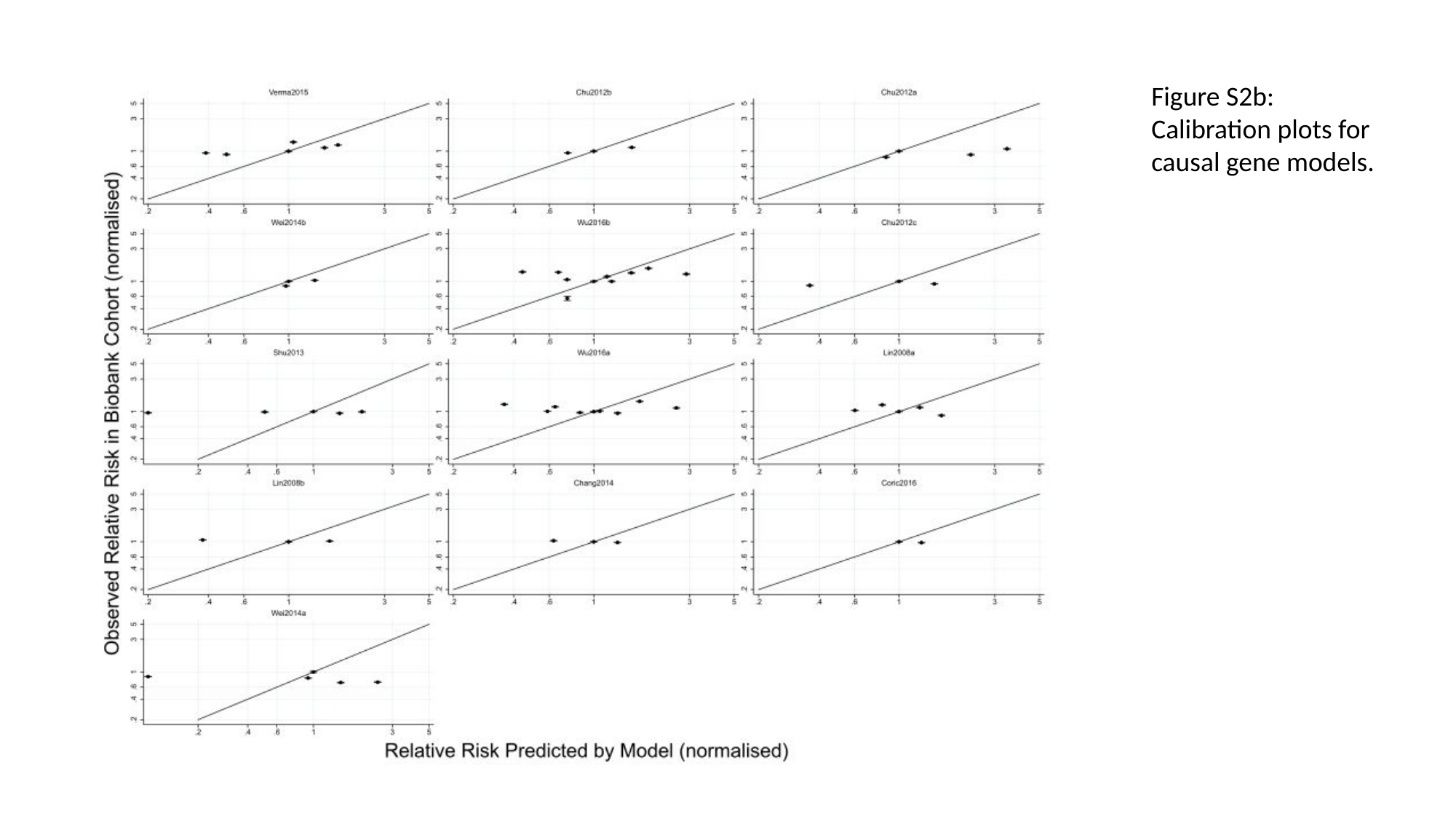

Figure S2b: Calibration plots for causal gene models.

## Slide 7
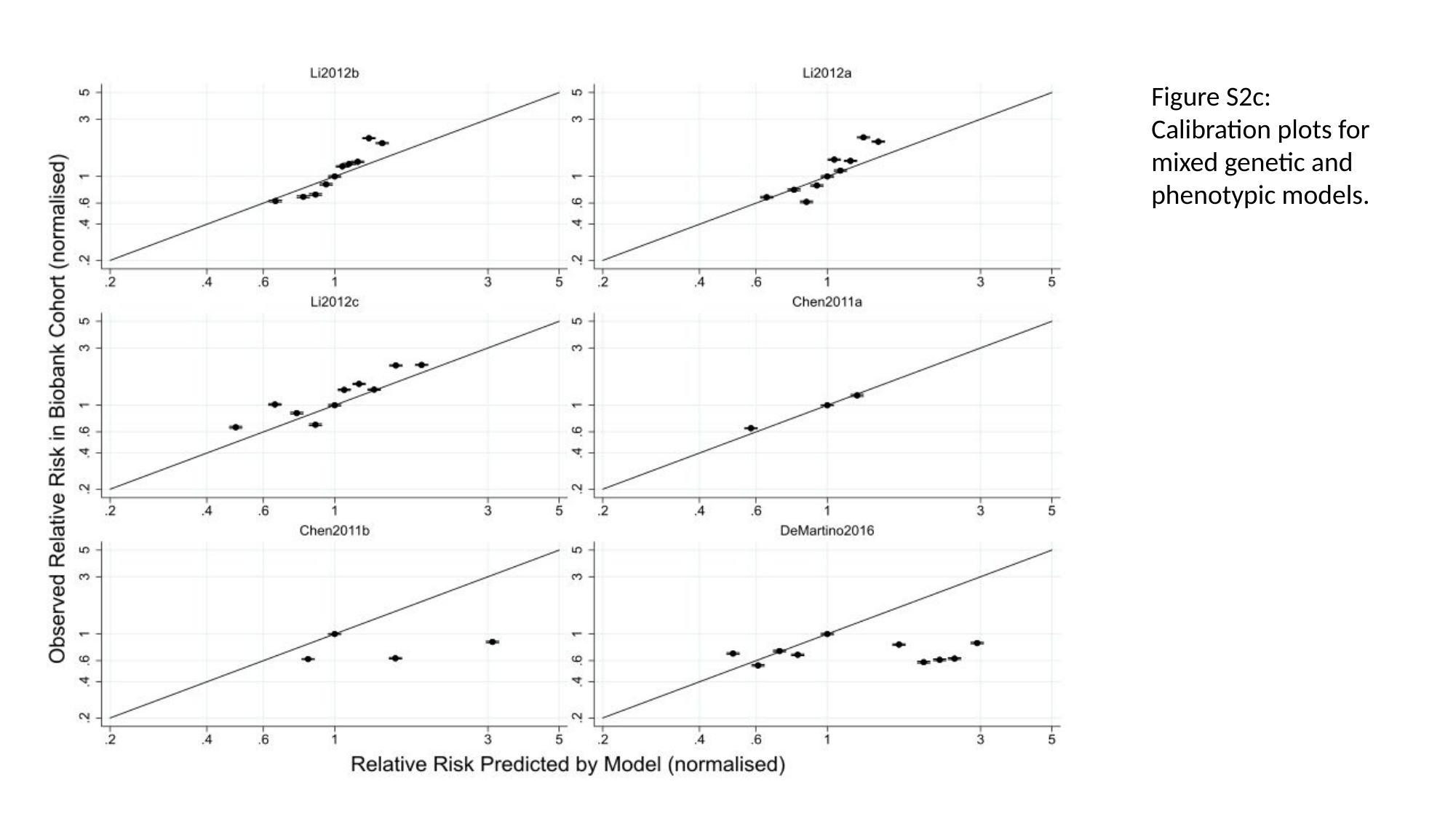

Figure S2c: Calibration plots for mixed genetic and phenotypic models.

## Slide 8
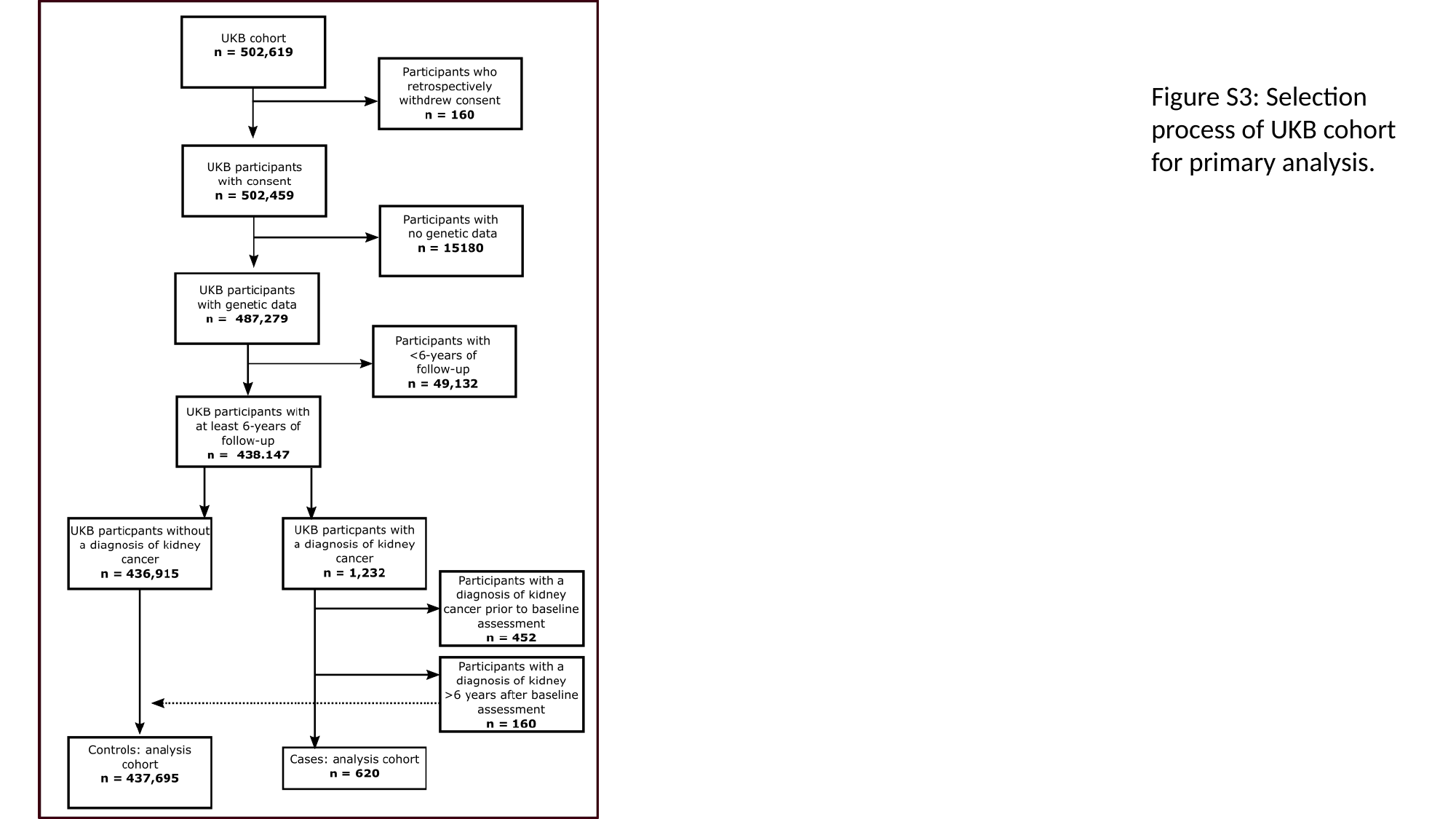

Figure S3: Selection process of UKB cohort for primary analysis.
