# Supplementary material for: The current state of genetic risk models for the development of kidney cancer: a review and validation
Source: BJU Int. 2022 May 7;130(5):550–61. doi: 10.1111/bju.15752 (PMC9790357; doi:10.1111/bju.15752)
Supplement: Supplementary file 2 — Table S1 . Details of included models. [file BJU-130-550-s001.docx]

**Supplementary Table 1 – Details of Included Models**

| **Model ID** | **Type of model** | **Considered (but not included) SNPs** | **Included SNPs** | **Smoking** | **Age** | **Sex** | **BMI** | **Hypertension** | **Other** |
| --- | --- | --- | --- | --- | --- | --- | --- | --- | --- |
| Arjumand2012^†^ | Logistic regression |  | rs10735810  rs1544410 | x |  | x | x | x |  |
| Arjumand2012^†^ | Combined odds ratio |  | rs10735810  rs1544410 |  |  |  |  |  |  |
| Chang2014 | Combined odds ratio (haplotype distribution analysis) | rs1997623  rs12672038  rs3757733  rs3807992 | rs3807987^β^  rs7804372 |  |  |  |  |  |  |
| Chen2011a | Combined odds ratio (logistic regression analysis) |  | rs1048943^β^ | x |  |  |  |  |  |
| Chen2011b | Combined odds ratio (logistic regression analysis) |  | rs4646903 | x |  |  |  |  |  |
| Chu2012a | Combined odds ratio (logistic regression analysis) | rs2243250  IL-4R Ser478Pro  IL-4R Gln551Arg | rs1805010  rs1900925 |  |  |  |  |  |  |
| Chu2012b | Combined odds ratio (logistic regression analysis) |  | rs1805010  rs20541 |  |  |  |  |  |  |
| Chu2012c | Combined odds ratio (logistic regression analysis) |  | rs1900925  rs20541 |  |  |  |  |  |  |
| Coric2017 | Polygenic risk score (sum of risk-carrying alleles) |  | rs1065411 ^α^  rs2266637 ^αβ^  rs3957358  rs1695 |  |  |  |  |  |  |
| DeMartino2016 | Logistic regression |  | rs2736098  rs2736100 rs7726159  rs2853677  rs13172201  rs10069690 | x | x | x | x |  | **MNS16A (minisatellite tandem repeat)** |
| Hsueh2017a^†^ | Logistic regression (combined effects analysis) |  | rs1799782 |  |  |  |  |  | **Urinary 8-OHdG levels** |
| Hsueh2017b^†^ | Logistic regression (combined effects analysis) |  | rs1799782 |  |  |  |  |  | **Urinary total arsenic** |
| Hsueh2018a | Logistic regression (combined effects analysis) |  | rs182052 | x | x | x | x | x | Education level  Alcohol Consumption  Diabetes  **Urinary creatinine levels**  **Urinary total arsenic** |
| Hsueh2018b | Logistic regression (combined effects analysis) |  | rs2241766 | x | x | x | x | x | Education level  Alcohol Consumption  **Urinary creatinine levels**  **Urinary total arsenic** |
| Hsueh2018c | Logistic regression (combined effects analysis) |  | rs1501299 | x | x | x | x | x | Education level  Alcohol Consumption  Diabetes  **Urinary creatinine levels**  **Urinary total arsenic** |
| Hsueh2018d | Logistic regression (combined effects analysis) |  | rs182052 |  | x | x | x | x | Education level  Alcohol Consumption  Diabetes  **Urinary creatinine levels**  **Urinary total arsenic** |
| Li2012a | Logistic regression |  | rs8106922 | x | x | x | x | x | Education, ethnicity |
| Li2012b | Logistic regression (using semiparametric maximum likelihood estimator) |  | rs8106922 | x | x | x | x | x | Education, ethnicity |
| Li2012c | Logistic regression (using pseudo-semiparametric maximum likelihood estimator) |  | rs8106922 | x | x | x | x | x | Education, ethnicity |
| Lin2008a | Polygenic risk score (sum of risk-carrying alleles) |  | rs321986  rs1052559 rs1799793 rs2020955^β^ rs17655 rs2228001 rs2228000 rs1805329 rs2266690 rs2228526 rs4253211^αβ^  rs1800975 |  |  |  |  |  | **XPC (xeroderma pigmentosum complementation group C) intron 9 (PAT) – in linkage disequilibrium with** **rs2228001** |
| Lin2008b | Combined odds ratio (CART Analysis) | rs1052559  rs2020955  rs17655  rs2228000  rs2266690 | rs1800975  rs1799793  rs2228001  rs1805329  rs321986  rs2228526  rs4253211 ^αβ^ |  |  |  |  |  |  |
| Machiela2017a | Polygenic Risk Score (weighted sum of risk-carrying alleles, developed for outcome of RCC) |  | rs10936599  rs11125529  rs2736100  rs3027234  rs6772228^β^  rs755017  rs7675998  rs8105767  rs9420907 |  |  |  |  |  |  |
| Machiela2017b | Polygenic Risk Score (weighted sum of risk-carrying alleles, developed for outcome of longer genetically inferred telomere length) |  | rs10936599  rs11125529  rs2736100  rs3027234  rs6772228^β^  rs755017  rs7675998  rs8105767  rs9420907 |  |  |  |  |  |  |
| Scelo2016 | Polygenic Risk Score (weighted sum of risk-carrying alleles) | 29 SNPs (see supplementary tables) | rs7105934^β^  rs4765623  rs718314  rs11894252  rs12105918^β^  rs6470588  rs4381241  rs67311347  rs10936602  rs2241261  rs11813268  rs74911261^β^  rs4903064 |  |  |  |  |  |  |
| Shu2013 | Polygenic risk score (sum of risk-carrying alleles) | 190 SNPs (see supplementary table) | rs4132509  rs3766673  rs12031994  rs4430311  rs1058304  rs2345994 |  |  |  |  |  |  |
| Verma2015 | Combined odds ratio (logistic regression analysis) | rs2910164  rs895819  rs12976445 | rs11614913  rs3746444 |  |  |  |  |  |  |
| Wei2014a | Polygenic risk score (sum of risk-carrying alleles) | 53 SNPs (see supplementary table) | rs743409  rs9607241  rs6773576  rs406271  rs10982724 |  |  |  |  |  |  |
| Wei2014b | Combined odds ratio (CART Analysis) | rs743409  rs406271 | rs9607241  rs6773576  rs10982724 |  |  |  |  |  |  |
| Wu2016a | Polygenic Risk Score (weighted sum of risk-carrying alleles normalized by genotype frequency in target population) | rs1326889  rs9679290  rs4953346  rs12617313  rs1492078  rs2010963  rs2279776  rs17512051  rs895819 | rs10054504  rs7023329  rs718314 |  |  |  |  |  |  |
| Wu2016b | Polygenic Risk Score (weighted sum of risk-carrying alleles normalized by genotype frequency in target population) |  | rs10054504  rs7023329  rs1049380 |  |  |  |  |  |  |
| Graff2021 | Polygenic Risk Score (weighted sum of risk-carrying alleles) |  | rs4381241 rs3845536 rs11894252 rs4953346 rs6755594 rs12105918 rs67311347 rs10936602 rs234043  rs7697932 rs2241261 rs11813268 rs7105934 rs74911261 rs718314  rs4765623 rs8007348 rs4903064  rs714024 |  |  |  |  |  |  |
| Shi2019a | Polygenic Risk Score (weighted sum of risk-carrying alleles normalized by genotype frequency in development population) |  | rs4381241 rs3845536 rs7579899 rs12105918 rs10936602 rs2241261 rs11813268 rs7105934 rs74911261 rs718314 |  |  |  |  |  |  |
| Shi2019b | Polygenic Risk Score (weighted sum of risk-carrying alleles normalized by genotype frequency in target population (UK Biobank) |  | rs4381241 rs3845536 rs7579899 rs12105918 rs10936602 rs2241261 rs11813268 rs7105934 rs74911261 rs718314 |  |  |  |  |  |  |
| Fritsche2018 | Polygenic Risk Score (weighted sum of risk-carrying alleles) |  | rs2495478  rs3755132  rs807624  rs1027643  rs790356  rs2283873  rs5955543 |  |  |  |  |  |  |
| Fritsche2020a | Polygenic Risk Score (weighted sum of risk-carrying alleles) |  | rs4381241 rs3755132 rs13086983 rs10936602 rs1027643 rs2241261 rs11813268 rs790356 rs74911261 rs4903064 rs2283873 rs5955543 |  |  |  |  |  |  |
| Fritsche2020b | Polygenic Risk Score (weighted sum of risk-carrying alleles) |  | rs4381241 rs3755132 rs13086983 rs10936602 rs1027643 rs2241261 rs11813268 rs790356 rs74911261 rs4903064 rs2283873 rs5955543 |  |  |  |  |  |  |
| Kachuri2020 | Polygenic Risk Score (inverse variance weighted sum of risk-carrying alleles) |  | rs4381241 rs3845536 rs11894252  rs4953346 rs6755594 rs12105918 rs67311347 rs10936602 rs234043  rs7697932 rs2241261 rs11813268 rs7105934 rs74911261 rs718314  rs4765623 rs8007348 rs4903064  rs714024 |  |  |  |  |  |  |
| Jia2020 | Polygenic Risk Score (weighted sum of risk-carrying alleles) |  | rs4381241 rs3845536 rs7579899 rs12617313 rs12105918 rs67311347 rs10936602 rs2241261 rs6470588 rs11813268 rs7105934 rs74911261 rs718314  rs4765623 rs4903064 |  |  |  |  |  |  |

^α^Imputation score <0.90, ^β^MAF <1%,

^†^model not validated within this study

**Text in bold indicates variables not available for the UK Biobank cohort**

**Supplementary Table 2 – Included Studies and their Development Populations**

| **First author** | **Year** | **Design^ǂ^** | **Outcome ^χ^** | **Country** | **Ethnicity** | **Sex (%male)** | | **Age (µ±σ)** | | **Population Size** | | **SNP selection** |
| --- | --- | --- | --- | --- | --- | --- | --- | --- | --- | --- | --- | --- |
|  |  |  |  |  |  | **Cases** | **Controls** | **Cases** | **Controls** | **Controls** | **Cases** |  |
| Arjumand | 2012 | CC | RCC | Indian | Asian (Indian) ^α^ | 71.4 | | 54.2 (se:0.97) | 54.0 (se:0.92) | 196 | 250 | Biological and epidemiological data had previously suggested a link between vitamin D levels and cancer development. Two polymorphisms from the VDR gene that mediates vitamin D activity were selected. |
| Chang | 2014 | CC | RCC | Taiwan | Asian (Chinese)^α^ | 64 | | 58.8 (11.7) | 58.3 (11.5) | 580 | 92 | Several SNPs from Cav-1 gene (Cav-1 has been associated with carcinogenesis) were tested for significant association with RCC in study population to determine inclusion. |
| Chen | 2011 | CC | RCC | China | Asian (Chinese)^α^ | 62 | 60 | 53.6 (10.2) | 52.7 (11.3) | 350 | 181 | CYPA1 polymorphisms previously shown to be associated with solid cancers. |
| Chu | 2012 | CC | RCC | China | Asian (Chinese)^α^ | 63.9 | | 56.9 (12.0) | 56.8 (11.9) | 623 | 620 | SNPs previously shown to be associated with immunoregulatory responses (from interleukin-4 and -13 genes) were tested for significant association with RCC in study population to determine inclusion. |
| Coric | 2017 | CC | RCC | Serbia | White | 66 | 64 | 59.3 (11.5) | 60.8 (11.5) | 326 | 305 | SNPs encoding for the glutathione S-transferase family (associated with differential susceptibility to stresses). |
| de Martino | 2016 | CC | ccRCC | Austria | Not given | 70.4 | 71.7 | 63.2 (12.7) | 62.7 (15.8) | 420 | 243 | SNPs on hTERT gene previously shown to be associated with malignancies or telomere length. |
| Hsueh | 2017 | CC | RCC | Taiwan | Asian (Chinese)^α^ | 63.3 | | 57.7 (se:1.03) | 59.8 (se:0.64) | 180 | 360 | Previous studies had shown that DNA repair polymorphism, urinary arsenic concentrations, 8-OHdg levels and RCC were linked. |
| Hsueh | 2018 | CC | RCC | Taiwan | Asian (Chinese)^α^ | 67.9 | | 58.8 (se:0.66) | 58.3 (se:0.66) | 389 | 389 | Previously reported interactions between adiponectin levels (a protein coded by the adiponectin gene), obesity, arsenic exposure and higher risk of RCC. |
| Li | 2012 | CC | KCa | USA | Black (708) and White (604) | 57.2 | 63.2 | <51: 12.6%  51-55:21.3%  56-62:31.2%  63-68:25.0%  >68: 9.9% | <51: 12.4%  51-55:21.4%  56-62:29.0%  63-68:27.5%  >68: 9.8% | 747 | 790 | SNP found to be associated with risk of kidney cancer in previous analysis in study population. |
| Lin | 2008 | CC | RCC | USA | White | 66.5 | 61.2 | 59.4 (10.8) | 59.7 (10.8) | 335 | 325 | SNPs previously reported to have potential significance in cancer association studies. |
| Machiela | 2017 | GWAS | RCC and telomere length | SNPs identified and weights calculated in GWAS studies: Scelo2017 | | | | | | | | SNPs identified in previous GWAS studies as being associated with leukocyte telomere length (p<5x10^8^). |
| Scelo | 2017 | GWAS | RCC | SNPs identified and weights calculated in GWAS studies: Henrion2013, Purdue2011 and Wu2012. Additionally, other GWAS results were reported in this study (Scelo2017). | | | | | | | | SNPs identified in GWAS scans as associated with RCC, results of both previous and current scans included. |
| Shu | 2012 | CC | RCC | USA | White (non-Hispanic) | 66.2 | 64.4 | 59.3 (10.7) | 58.9 (9.3) | 593 | 577 | 190 SNPs (157 original SNPs and 33 proxy SNPs) from 22 genes in the mTOR (mammalian target of rapamycin) pathway were analysed. 28 SNPs were significantly associated with RCC risk after adjustments (*P* < .05). Six SNPs located in the *AKT3* gene remained statistically significant using the criterion *Q* < .05 and were included in the model. |
| Verma | 2015 | CC | RCC | North India | Asian (Indian) | 81.0 | 89.8 | 49.9 (11.4) | 55.1 (10.1) | 225 | 100 | SNPs in miRNA genes previously associated with solid cancers were tested for significant association with RCC in study population to determine inclusion. |
| Wei | 2014 | CC | RCC | USA | White (non-Hispanic) | 68.1 | 77.4 | 59.7 (10.7) | 63.2 (10.9) | 1156 | 894 | SNPs (n=100) in were screened for effect on miRNA-binding sites, then tested for association with RCC risk. |
| Wu | 2016 | CC | RCC | China | Asian (Chinese)^α^ | 66.5 | 64.8 | 55.9 (12.5) | 51.2 (9.7) | 1130 | 346 | SNPs shown to be associated with RCC in other studies were tested for significant association with RCC in study population to determine inclusion. |
| Graff | 2021 | GWAS | KCa | SNPs identified and weights calculated in GWAS studies: Henrion2015, Scelo2017 | | | | | | | | Variants were identified from existing published GWAS for RCC. SNPs identified in populations of at least 70% European ancestry were included. |
| Shi | 2019 | GWAS | KCa | SNPs identified and weights calculated in GWAS studies: Scelo2017, Henrion2015, Purdue2011, Henrion2012 and Wu2012 | | | | | | | | Cancer-specific risk-associated SNPs were extracted from GWAS papers published prior to 1^st^ July 2018. They were included in the polygenic risk score if the GWAS studies used Caucasian individuals, had at least 1000 cases and 1000 controls, p<5*10^-8 and linkage disequilibrium r^2 <0.2 between any two SNPs. |
| Fritsche | 2018 | GWAS | KCa | SNPs identified and weights calculated in GWAS study: Turnbull2012 | | | | | | | | Previously reported GWAS variants from the NHGRO-EBI Catalog (up to June 2017) – European ancestry only. |
| Fritsche | 2020 | GWAS | KCa | SNPs identified and weights calculated in GWAS studies: Zhou2018, Turnbull2012 and Scelo2017 | | | | | | | | Extracted variants from GWAS summary statistics from three different sources: (1) merged genome-wide significant association signals published in the NHGRI EBI GWAS Catalog45 if available; (2) large cancer GWAS meta-analysis if available; and (3) publicly available GWAS summary statistics of phenome 3 genome screening efforts of the UK Biobank data. |
| Kachuri | 2020 | GWAS | KCa | SNPs identified and weights calculated in GWAS studies: Scelo2017 and Henrion2015 | | | | | | | | Extracted previously associated variants by searching the National Human Genome Research Institute-European Bioinformatics Institute Catalog of published GWAS. Additional relevant studies were identified by searching the reference section of included studies. Autosomal variants with MAF >= 0.01 and p<5*10^-8 identified in populations of at least 70% European ancestry were included. |
| Jia | 2020 | GWAS | KCa | SNPs identified and weights calculated in GWAS studies: Henrion2015, Purdue2011, Han2012, Henrion2012, Scelo2017, Gudmundsson2013 and Wu2012. | | | | | | | | Extracted genetic variants identified by previous GWAS in association with the risk of kidney cancer by reviewing the GWAS catalog and previous PubMed publications. Single-nucleotide polymorphisms (SNPs) specifically associated with the risk of a specific subtype of kidney cancer were not included in the analysis. Cancer risk variants on the X chromosome, reported exclusively from non-European populations, or in high linkage disequilibrium (r2 >= 0.2 in data of European ancestry in the 1000 Genomes project database), were also excluded in this study. For some previously reported risk variants that were not available in the data from UK Biobank, SNPs in high linkage disequilibrium (r2 >= 0.85) were selected for the study. |

**^α^Ethnicity assumed to be Asian (Chinese) based on study location**

**^β^Ethnicity assumed to be White based on “European ancestry/background” statement**

**^†^ The mean and standard deviation of the age of the development population are given, unless otherwise stated, e.g. se (standard error of the mean)**

**^χ^ RCC (renal cell carcinoma), ccRCC (clear-cell renal cell carcinoma), KCa (kidney cancer, all types),**

**^ǂ^CC- case-control study design, SNPs identified through GWAS (genome-wide association studies)**

**SNP (single nucleotide polymorphism)**

**Supplementary Table 3 – Previously Published External Validations**

| **First Author, Year** | **Disease (Outcome) ^χ^** | **Country** | **Ethnicity** | **Cases** | **Controls** | **Named cohorts^†^** | **Performance Measures** |
| --- | --- | --- | --- | --- | --- | --- | --- |
| Graff, 2021 | KCa | USA, UK | European ancestry | 1341 | 410354 | GERA, UKB | Effect size/sd, OR: 1.21 (lb/ub: 1.14-1.26) |
| Shi, 2019 | KCa | USA | Caucasians only | 453 | 13427 | TCGA, eMERGE | Mean score for cases, RR: 1.09 (95% CI: 1.06-1.12) |
| Fritsche, 2018 | KCa | USA | European ancestry | 613 | 26748 | MGI | Ratio of highest to lowest decile, OR: 0.98 (0.77-1.3) |
| Fritsche, 2020 | KCa | USA, UK | Recent European ancestry, White British | 1116 | 445839 | MGI, UKB | Discrimination, AUC: 0.527 (0.492-0.540)  OR: 1.15 (1.06-1.25) |
| Kachuri, 2020 | KCa | UK | Predominately European ancestry | 612 | 413141 | UKB | Only given for combined model incorporating phenotypic factors and PRS. |
| Jia, 2020 | KCa | UK | Europeans only | 545 | 400267 | UKB | Discrimination, AUC: 0.567 (95% CI: 0.543-0.591)  Mean (sd) of score, cases: 2.257 (0.41), controls: 2.171 (0.40) |

**^χ^KCa – kidney cancer (all types)**

**^†^GERA = Genetic Epidemiology Research on Adult Health and Aging , UKB = UK Biobank, TCGA = Cancer Genome Atlas, MGI = Michigan Genomics Initiative**

**Supplementary Table 4 (a&b) – Use of UKB phenotypic variables**

| **Variables** | **UKB Variables Used in Models** | **Method** | **% Missing** |
| --- | --- | --- | --- |
| **Age** | Age at Baseline | Direct match. Either continuous (deMartino2016) or recoded into categories (Li2012, 4 categories). | 0% |
| **Sex** | Sex (male or female) | Direct match (deMartino2016, Li2012). | 0% |
| **BMI** | BMI (constructed from height and weight measured at the baseline assessment) | Direct match. Continuous measure of BMI was recoded as a categorical variable (Li2012 (4 categories), deMartino2016 (2 categories)). | 0.36% |
| **Smoking** | Smoking status Number of cigarettes consumed daily (current) Number of cigarettes consumed daily (former) Age started smoking Age stopped smoking  Smoking breaks | Smoking status (current, former, never) was recoded to never/ever smoker (Li2012, Chen2011).  In the deMartino2016 model, a measure of pack years is required. Duration of smoking (calculated from age at start/stop and information about smoking breaks) is combined with intensity (number of cigarettes consumed daily). Given the high proportion of missing data in the age and intensity variables, the composite pack-year variable is missing for 33% of current and former smokers. Multiple imputation (details in supplementary methods) was used to manage this missing data. | 0.36% (smoking status)  33% (pack years – before imputation) |
| **Education Level** | Education level Age education ended | Age education ended was used to estimate the number of years each individual had been in full time education (assuming beginning education aged 5). Where this information was missing, the education level reported by the participant was used to obtain an estimate of education length. This was then recoded to the categorical variables in Li2012 (<=11years, 11-13years, >=13years of education). | 1.64% |
| **High Blood Pressure** | Self-reported heart issues (diagnosed by a doctor) | Coded as hypertension, was used a direct match in Li2012 to normal and high blood pressure | 0.22% |
| **SNP data** | Full genotype information available for almost all members of cohort. | Where possible imputed continuous SNP values were used for the models (Scelo, Machiela, Wu).  In some models it was necessary to use categorical versions of the SNP data. The SNPs variables were recoded accordingly, 0 – 0.1 => 0, 0.9-1.1=> 1 and 1.9 – 2 => 2, all other values were coded as missing. | See supplementary file with table of SNPs |

| **Variables** | **UKB Variables Used in Sensitivity Analysis** | **Method** | **% Missing** |
| --- | --- | --- | --- |
| **Sex** | Sex (male or female) | Sensitivity analyses were run for all male and female members of the cohort separately. | 0% |
| **Ethnicity** | Ethnicity (self-reported at baseline assessment as White/European, Mixed, South Asian, Black and Other) | In one sensitivity analysis, only participants who self-reported as white were included. | 0.33% |

**SNPs (single nucleotide polymorphisms)**

**Supplementary Table 5 – Primary Analyses (external validation in UK Biobank): model accuracy in deciles**

| **Models** | **Whole Cohort** | | | **Highest 10%** | | | | **Lowest 10%** | | | |
| --- | --- | --- | --- | --- | --- | --- | --- | --- | --- | --- | --- |
|  | **Cohort** | **Cases** | **PPV (%)** | **Sens (%)** | **Spec (%)** | **PPV (%)** | **NPV (%)** | **Sens (%)** | **Spec (%)** | **PPV (%)** | **NPV (%)** |
| **DeMartino2016** | 368911 | 516 | 0.140 | 11.6 | 90.2 | 0.165 | 99.9 | 9.7 | 90.0 | 0.135 | 99.9 |
| **Li2012a** | 425903 | 608 | 0.143 | 16.1 | 90.3 | 0.238 | 99.9 | 6.4 | 88.8 | 0.082 | 99.8 |
| **Li2012b** | 425903 | 608 | 0.143 | 16.3 | 90.0 | 0.233 | 99.9 | 5.8 | 89.3 | 0.077 | 99.8 |
| **Li2012c** | 425903 | 608 | 0.143 | 16.9 | 90.1 | 0.243 | 99.9 | 5.3 | 89.8 | 0.074 | 99.8 |
| **Machiela2017a** | 435572 | 615 | 0.141 | 9.6 | 90.1 | 0.137 | 99.9 | 7.6 | 90.0 | 0.108 | 99.9 |
| **Machiela2017b** | 435572 | 615 | 0.141 | 10.9 | 90.0 | 0.154 | 99.9 | 8.0 | 90.0 | 0.112 | 99.9 |
| **Scelo2016** | 435572 | 615 | 0.141 | 14.0 | 90.0 | 0.197 | 99.9 | 6.8 | 90.0 | 0.096 | 99.9 |
| **Wu2016b** | 435572 | 615 | 0.141 | 6.3 | 94.0 | 0.148 | 99.9 | 11.4 | 89.9 | 0.159 | 99.9 |
| **Graff2021** | 435572 | 615 | 0.141 | 11.9 | 90.0 | 0.168 | 99.9 | 8.1 | 90.0 | 0.115 | 99.9 |
| **Shi2019a** | 435572 | 615 | 0.141 | 14.3 | 90.0 | 0.202 | 99.9 | 8.5 | 90.0 | 0.119 | 99.9 |
| **Shi2019b** | 435572 | 615 | 0.141 | 14.3 | 90.0 | 0.203 | 99.9 | 8.1 | 90.0 | 0.115 | 99.9 |
| **Fritsche2021a*** | 434969 | 614 | 0.141 | 11.7 | 90.0 | 0.166 | 99.9 | 7.7 | 90.0 | 0.108 | 99.9 |
| **Fritsche2021b*** | 434969 | 614 | 0.141 | 11.7 | 90.0 | 0.166 | 99.9 | 7.7 | 90.0 | 0.108 | 99.9 |
| **Kachuri2020** | 435572 | 615 | 0.141 | 12.2 | 90.0 | 0.172 | 99.9 | 7.2 | 90.0 | 0.101 | 99.9 |
| **Jia2020** | 435572 | 615 | 0.141 | 13.2 | 90.0 | 0.186 | 99.9 | 6.7 | 90.0 | 0.094 | 99.9 |

***Some of the SNPs identified in a genome-wide association study using UK Biobank participants, so not a true external validation**

**Sens – sensitivity, spec – specificity, PPV – positive predictive value, NPV – negative predictive value**

**Supplementary Table 6: Primary Analyses (external validation in UK Biobank): alternative measures of discrimination**

| **Model Name** | **Type** | **Cases** | **Cohort** | **MSS Cases (sd)** | **MSS Controls (sd)** | **OR per sd (se)** |
| --- | --- | --- | --- | --- | --- | --- |
| **Lin2008a** | Causal Genes | 615 | 435572 | 0.002194 (1.020236) | -0.000003 (0.999972) | 1.002 (0.040) |
| **Lin2008b** | Causal Genes | 605 | 426092 | -0.011475 (1.006502) | 0.000016 (0.999992) | 0.989 (0.040) |
| **Chu2012b** | Causal Genes | 610 | 433036 | 0.063325 (1.042099) | -0.000089 (0.999938) | 1.063 (0.041) |
| **Chu2012a** | Causal Genes | 610 | 431465 | 0.044742 (1.032686) | -0.000063 (0.999953) | 1.045 (0.041) |
| **Chu2012c** | Causal Genes | 612 | 432797 | 0.027445 (1.001553) | -0.000039 (0.999998) | 1.028 (0.041) |
| **Shu2013** | Causal Genes | 615 | 435572 | 0.022726 (1.011035) | -0.000032 (0.999985) | 1.023 (0.041) |
| **Wei2014b** | Causal Genes | 593 | 415664 | 0.011869 (0.89002) | -0.000017 (1.000149) | 1.012 (0.040) |
| **Chang2014** | Causal Genes | 613 | 433109 | -0.028735 (0.996761) | 0.000041 (1.000005) | 0.971 (0.040) |
| **Wei2014a** | Causal Genes | 615 | 435572 | -0.028166 (0.977862) | 0.00004 (1.000031) | 0.972 (0.040) |
| **Verma2015** | Causal Genes | 604 | 428885 | 0.092375 (0.98855) | -0.00013 (1.000011) | 1.098 (0.045) |
| **Wu2016b** | Causal Genes | 615 | 435572 | 0.053198 (1.021008) | -0.000075 (0.999969) | 1.052 (0.040) |
| **Wu2016a** | Causal Genes | 615 | 435572 | 0.025995 (1.020042) | -0.000037 (0.999972) | 1.026 (0.040) |
| **Coric2016** | Causal Genes | 615 | 435572 | -0.035398 (1.012099) | 0.00005 (0.999983) | 0.966 (0.055) |
| **Scelo2017** | PRS | 615 | 435572 | 0.172526 (1.026564) | -0.000244 (0.999942) | 1.189 (0.051) |
| **Machiela2017a** | PRS | 615 | 435572 | 0.069564 (0.965978) | -0.000098 (1.000045) | 1.072 (0.043) |
| **Machiela2017b** | PRS | 615 | 435572 | 0.072719 (0.991951) | -0.000103 (1.000009) | 1.076 (0.044) |
| **Fritsche2018b** | PRS | 614 | 434969 | -0.030 (0.981) | 0.000043 (1.00003) | 0.97 (0.039) |
| **Fritsche2018a** | PRS | 614 | 434969 | -0.034 (1.010) | 0.000048 (0.99999) | 0.97 (0.040) |
| **Shi2019a** | PRS | 615 | 435572 | 0.152 (1.013) | -0.0002 (0.99997) | 1.164 (0.047) |
| **Shi2019b** | PRS | 615 | 435572 | 0.153 (1.013) | -0.0002 (0.99997) | 1.164 (0.047) |
| **Jia2020** | PRS | 615 | 435572 | 0.2 (1.005) | -0.0003 (0.99997) | 1.222 (0.049) |
| **Kachuri2020** | PRS | 615 | 435572 | 0.159 (0.991) | -0.0002 (0.999995) | 1.171 (0.047) |
| **Graff2021** | PRS | 615 | 435572 | 0.175 (1.007) | -0.0002 (0.99997) | 1.192 (0.048) |
| **Fritsche2020a*** | PRS | 614 | 434969 | 0.057 (1.057) | -0.0001 (0.99992) | 1.056 (0.041) |
| **Fritsche2020b*** | PRS | 614 | 434969 | 0.057 (1.057) | -0.0001 (0.99992) | 1.056 (0.041) |
| **Chen2011a** | Mixed | 612 | 434120 | 0.230154 (1.012157) | -0.000325 (0.999946) | 1.253 (0.050) |
| **Chen2011b** | Mixed | 612 | 434439 | 0.035245 (1.027696) | -0.00005 (0.999961) | 1.034 (0.040) |
| **Li2012b** | Mixed | 608 | 425903 | 0.389572 (0.972188) | -0.000557 (0.999932) | 1.49 (0.062) |
| **Li2012a** | Mixed | 608 | 425903 | 0.390133 (0.989294) | -0.000558 (0.999907) | 1.476 (0.060) |
| **Li2012c** | Mixed | 608 | 425903 | 0.377038 (1.041853) | -0.000539 (0.999838) | 1.421 (0.054) |
| **DeMartino2016** | Mixed | 516 | 368911 | 0.002172 (1.001062) | -0.000003 (1) | 1.002 (0.044) |

**MSS – mean standardised score, OR – Odds ratio, sd - standard deviation, se – standard error**

***Some of the SNPs identified in a genome-wide association study using UK Biobank participants, so not a true external validation**
