# Supplementary material for: The current state of genetic risk models for the development of kidney cancer: a review and validation
Source: BJU Int. 2022 May 7;130(5):550–61. doi: 10.1111/bju.15752 (PMC9790357; doi:10.1111/bju.15752)
Supplement: Supplementary file 3 — Table S7 . Details of the single nucleotide polymorphisms (SNPs) used in the analysis. [file BJU-130-550-s004.pdf]

Rs Number	SNP	Alternative Name	Effect Allele	MAF (UKB)	Imputation Score	Study
rs4253211	10:50678317_C_T	ERCC6 Arg1230Pro	C	9.76E-05	0.54	Lin2008
rs2266637	22:24376845_C_T	GSTT1	A (T)	1.11E-04	0.76	Coric2017
rs2020955	16:14038659_T_C	XPF Pro662Ser	T	0.01	0.93	Lin2008
rs1048943	15:75012985_T_C	CYP1A1 Ile/Val	C	0.03	0.98	Chen2011
rs5955543	X:17698397_A_G		G	0.02	1.00	Fritsche2018, Fritsche2021
rs2283873	22:31013296_G_A		A	0.03	1.00	Fritsche2018, Fritsche2021
rs13086983	3:40574734_C_T		G	0.04	1.00	Fritsche2021
rs74911261	11:108357137_G_A		A	0.02	1.00	Scelo2016, Shi2019, Kachuri2020, Jia2020, Graff2021, Fritsche2021
rs1065411	1:110233138_G_C	GSTM1	G	0.33	0.71	Coric2017
rs13172201	5:1271661_T_C		T	0.25	0.90	DeMartino2016
rs2853677	5:28837505_A_C		G	0.35	0.96	DeMartino2016
rs6772228	3:58376019_T_A		T	0.05	0.96	Machiela2017
rs4765623	12:125320850_C_T		T	0.34	0.96	Scelo2016, Kachuri2020, Jia2020, Graff2021
rs321986	7:78298061_G_C	ERCC1 3'UTR	C	0.37	0.97	Lin2008
rs12105918	2:145208193_T_C		C	0.06	0.97	Scelo2016, Shi2019, Kachuri2020, Jia2020, Graff2021
rs6773576	3:45117173_A_G	CDCP1 (gene)	A	0.19	0.97	Wei2014
rs2736098	5:1294086_C_T		G (C)	0.28	0.98	DeMartino2016
rs8007348	14:73272730_C_A		A	0.26	0.98	Kachuri2020, Graff2021
rs6470588	8:128889371_A_C		C	0.48	0.98	Scelo2016, Jia2020
rs67311347	3:40533243_G_A		G	0.30	0.98	Scelo2016, Kachuri2020, Jia2020, Graff2021
rs2241261	8:22876739_C_T		T	0.48	0.98	Scelo2016, Shi2019, Kachuri2020, Jia2020, Graff2021, Fritsche2021
rs4430311	1:244015993_C_T		G (C)	0.33	0.98	Shu2013
rs1049380	12:26489544_G_T		C (G)	0.27	0.98	Wu2016
rs3807987	7:116179834_G_A	Cav-1 G14713A	A	0.07	0.99	Chang2014
rs7804372	7:116194228_T_A	Cav-1 T29107A	A	0.25	0.99	Chang2014
rs4646903	15:75011641_A_G	CYP1A1 MspI	G	0.11	0.99	Chen2011
rs1900925	11:132140629_A_G	IL-13 C-1055T	T	0.34	0.99	Chu2012
rs1805010	16:27356203_A_G	IL-4R Ile50Val	T	0.45	0.99	Chu2012
rs3957357	6:52668687_A_G	GSTA1	G	0.12	0.99	Coric2017
rs12617313	2:46559776_A_T		T	0.48	0.99	Jia2020
rs7697932	4:101005318_A_G		G	0.33	0.99	Kachuri2020, Graff2021
rs6755594	2:46589295_A_G		G	0.38	0.99	Kachuri2020, Graff2021
rs714024	22:47013535_C_T		G	0.40	0.99	Kachuri2020, Graff2021
rs8106922	19:45401666_A_G		G	0.38	0.99	Li2012
rs1805329	9:110084328_C_T	RAD23B Ala249Val	G (C)	0.19	0.99	Lin2008
rs1800975	9:100459578_T_C	XPA 5'UTR	G (C)	0.32	0.99	Lin2008

rs755017	20:62421622_A_G		G	0.13	0.99	Machiela2017
rs11125529	2:54475866_C_A		A	0.14	0.99	Machiela2017
rs7675998	4:164007820_A_G		G	0.21	0.99	Machiela2017
rs3027234	17:8136092_C_T		C	0.22	0.99	Machiela2017
rs8105767	19:22215441_A_G		G	0.29	0.99	Machiela2017
rs11894252	2:46533376_T_C		T	0.41	0.99	Scelo2016, Kachuri2020, Graff2021
rs11813268	10:105682296_C_T		T	0.16	0.99	Scelo2016, Shi2019, Kachuri2020, Jia2020, Graff2021, Fritsche2021
rs10936602	3:169536637_T_C		T	0.25	0.99	Scelo2016, Shi2019, Kachuri2020, Jia2020, Graff2021, Fritsche2021
rs4381241	1:50907438_T_C		C	0.47	0.99	Scelo2016, Shi2019, Kachuri2020, Jiia2020, Graff2021, Fritsche2021
rs3845536			C	0.37	0.99	Shi2019, Kachuri2020, Jia2020, Graff2021
rs12031994	1:243917309_T_C		A (T)	0.14	0.99	Shu2013
rs3766673	1:243720473_T_C		G (C)	0.18	0.99	Shu2013
rs2345994	1:243932275_T_C		A (T)	0.33	0.99	Shu2013
rs3746444	20:33578251_A_G	hsa-mir-499T/C	C (G)	0.18	0.99	Verma2015
rs11614913	12:54385599_C_T	hsa-mir-196a2C/T	T	0.41	0.99	Verma2015
rs10982724	9:118157729_T_C	DEC1 (gene)	G (C)	0.12	0.99	Wei2014
rs9607241	22:22104873_A_C	MAPK1 (gene)	C	0.41	0.99	Wei2014
rs743409	22:22129215_A_G	MAPK1 (gene)	A	0.48	0.99	Wei2014
rs2228001	3:14187449_G_T	XPC Lys939Gln	C (G)	0.39	1.00	Lin2008
rs20541	5:131995964_A_G	IL-13 Arg130Gln	A	0.18	1.00	Chu2012
rs1695	11:67352689_X_X	GSTP1	G	0.35	1.00	Coric2017
rs10069690	5:28837168_C_T		C	0.27	1.00	DeMartino2016
rs7726159	5:28837302_C_A		A	0.33	1.00	DeMartino2016
rs2736100	5:1286516_C_A		C	0.49	1.00	DeMartino2016, Machiela2017
rs807624	2:15782471_G_T		T	0.37	1.00	Fritsche2018
rs2495478	1:55512995_G_A	-	A	0.05	1.00	Fritsche2018
rs1027643	5:91893792_C_T	-	T	0.06	1.00	Fritsche2018, Fritsche2021
rs3755132	2:15729820_A_G		G	0.16	1.00	Fritsche2018, Fritsche2021
rs790356	11:83620787_A_G		G	0.49	1.00	Fritsche2018, Fritsche2021
rs234043	3:172313367_T_C		G	0.28	1.00	Kachuri2020, Graff2021
rs4953346	2:46558208_T_G		G	0.46	1.00	Kachuri2020, Graff2021
rs2228526	10:50678717_T_C	ERCC6 Met1097Val	G (C)	0.19	1.00	Lin2008
rs2266690/rs2230641	5:86695274_A_G	CCNH Val270Ala	C (G)	0.20	1.00	Lin2008
rs17655	13:103528002_G_C	XPG Asp110His	C	0.21	1.00	Lin2008
rs2228000	3:14199887_G_A	XPC Ala499Val	T (A)	0.24	1.00	Lin2008
rs1799793	19:45867259_C_T	XPD Asp312Asn	A (T)	0.32	1.00	Lin2008
rs1052559	19:45854919_T_G	XPD Lys751Gln/rs13181	C (G)	0.36	1.00	Lin2008

rs9420907	10:105676465_C_A		C	0.13	1.00	Machiela2017
rs10936599	3:169492101_C_T		C	0.24	1.00	Machiela2017
rs4903064	14:73279420_T_C		C	0.23	1.00	Scelo2016, Kachuri2020, Jia2020, Graff2021, Fritsche2021
rs7105934	11:69239741_G_A		G	0.09	1.00	Scelo2016, Shi2019, Kachuri2020, Jia2020, Graff2021
rs7579899			A	0.41	1.00	Shi2019, Jia2020
rs4132509	1:243943084_C_A		A	0.18	1.00	Shu2013
rs1058304	1:243664857_C_T		G (C)	0.24	1.00	Shu2013
rs406271	3:195776976_T_C	TFRC (gene)	G (C)	0.31	1.00	Wei2014
rs10054504	5:32000483_T_C		T	0.12	1.00	Wu2016
rs7023329	9:21816528_A_G	MTAP (gene)	A	0.49	1.00	Wu2016
rs718314	12:26453283_A_G		G	0.24	1.00	Wu2016, Scelo2016, Shi2019, Kachuri2020, Jia2020, Graff2021
rs10735810		VDR Fokl	unknown	not included in validation		Arjumand2012
rs1544410		BsmI	unknown	not included in validation		Arjumand2012
rs1799782		XRCC1 (Arg194TRrp)	T	not included in validation		Hsueh2017
rs182052		ADIPOQ (gene)	A	not included in validation		Hsueh2018
rs2241766		ADIPOQ (gene)	T	not included in validation		Hsueh2018
rs1501299		ADIPOQ (gene)	A	not included in validation		Hsueh2018
