# Supplementary material for: The current state of genetic risk models for the development of kidney cancer: a review and validation
Source: BJU Int. 2022 May 7;130(5):550–61. doi: 10.1111/bju.15752 (PMC9790357; doi:10.1111/bju.15752)
Supplement: Supplementary file 4 — Table S8 . Model discrimination (AUROC) in sensitivity analyses. [file BJU-130-550-s002.zip › BJU_15752_TableS8_SA_complete_cohort_across_all_models.pdf]

model	AUC	AUC_se	AUC_lb	AUC_ub	cohort	cases
Chang2014	0.48987	0.016773	0.456996	0.522744	163142	233
Chen2011a	0.613878	0.015648	0.583209	0.644548	163142	233
Chen2011b	0.563331	0.015488	0.532976	0.593686	163142	233
Chu2012a	0.519387	0.016606	0.48684	0.551933	163142	233
Chu2012b	0.526562	0.017633	0.492002	0.561122	163142	233
Chu2012c	0.497899	0.016541	0.465479	0.530319	163142	233
Coric2016	0.492806	0.016894	0.459695	0.525918	163142	233
DeMartino2016	0.493692	0.018393	0.457644	0.529741	163142	233
Li2012a	0.611212	0.018661	0.574638	0.647786	163142	233
Li2012b	0.61655	0.018521	0.580251	0.65285	163142	233
Li2012c	0.613214	0.018954	0.576065	0.650363	163142	233
Lin2008a	0.510279	0.019089	0.472864	0.547693	163142	233
Lin2008b	0.523124	0.017485	0.488854	0.557395	163142	233
Machiela2017a	0.518578	0.019184	0.480977	0.556178	163142	233
Machiela2017b	0.5176	0.019905	0.478587	0.556613	163142	233
Scelo2016	0.572423	0.018731	0.535712	0.609134	163142	233
Shu2013	0.524971	0.017406	0.490856	0.559085	163142	233
Verma2015	0.497281	0.018582	0.46086	0.533702	163142	233
Wei2014a	0.492006	0.017578	0.457554	0.526459	163142	233
Wei2014b	0.509403	0.015316	0.479384	0.539422	163142	233
Wu2016a	0.486475	0.019454	0.448345	0.524604	163142	233
Wu2016b	0.498123	0.01995	0.459022	0.537223	163142	233
Graff2021	0.57871	0.019215	0.54105	0.616371	163142	233
Shi2019a	0.555416	0.019008	0.518161	0.592672	163142	233
Shi2019b	0.554849	0.019023	0.517564	0.592134	163142	233
Fritsche2021a	0.51554	0.019245	0.47782	0.55326	163142	233
Fritsche2021b	0.51554	0.019245	0.47782	0.55326	163142	233
Kachuri2020	0.570594	0.019091	0.533176	0.608012	163142	233
Jia2020	0.578729	0.019002	0.541486	0.615972	163142	233
Fritsche2018a	0.490427	0.019788	0.451644	0.52921	163142	233
Fritsche2018b	0.503893	0.0184	0.467829	0.539957	163142	233
