# Supplementary material for: The current state of genetic risk models for the development of kidney cancer: a review and validation
Source: BJU Int. 2022 May 7;130(5):550–61. doi: 10.1111/bju.15752 (PMC9790357; doi:10.1111/bju.15752)
Supplement: Supplementary file 4 — Table S8 . Model discrimination (AUROC) in sensitivity analyses. [file BJU-130-550-s002.zip › BJU_15752_TableS8_SA_ethnicity_white_only.pdf]

model	AUC	AUC_se	AUC_lb	AUC_ub	cohort	cases
Chang2014	0.492252	0.010486	0.4717	0.512804	411591	602
Chen2011a	0.55385	0.010648	0.532981	0.574719	412277	601
Chen2011b	0.536202	0.010399	0.515821	0.556583	412424	601
Chu2012a	0.512225	0.010285	0.492067	0.532383	410085	599
Chu2012b	0.513578	0.010877	0.492259	0.534896	411213	599
Chu2012c	0.504252	0.010365	0.483937	0.524566	410835	601
Coric2016	0.491415	0.010908	0.470037	0.512793	413002	604
DeMartino2016	0.504384	0.012491	0.479901	0.528866	355578	511
Li2012a	0.608372	0.011566	0.585704	0.631041	405979	598
Li2012b	0.609482	0.011509	0.586924	0.63204	405979	598
Li2012c	0.607347	0.011632	0.584548	0.630147	405979	598
Lin2008a	0.502103	0.011545	0.479474	0.524731	413002	604
Lin2008b	0.496391	0.011189	0.474461	0.51832	405256	597
Machiela2017a	0.5237	0.011602	0.500961	0.54644	413002	604
Machiela2017b	0.522751	0.011823	0.499579	0.545923	413002	604
Scelo2016	0.546778	0.011734	0.52378	0.569775	413002	604
Shu2013	0.511876	0.011086	0.490147	0.533604	413002	604
Verma2015	0.525374	0.011549	0.502739	0.548009	407458	593
Wei2014a	0.490253	0.010988	0.468717	0.511789	413002	604
Wei2014b	0.509257	0.009828	0.489994	0.528519	395719	583
Wu2016a	0.509616	0.012204	0.485697	0.533534	413002	604
Wu2016b	0.513634	0.012341	0.489445	0.537823	413002	604
Graff2021	0.551768	0.011704	0.528828	0.574708	413002	604
Shi2019a	0.546403	0.011638	0.523593	0.569213	413002	604
Shi2019b	0.546305	0.011644	0.523484	0.569126	413002	604
Fritsche2021a	0.516135	0.01207	0.492478	0.539791	412399	603
Fritsche2021b	0.516135	0.01207	0.492478	0.539791	412399	603
Kachuri2020	0.54772	0.011689	0.52481	0.570631	413002	604
Jia2020	0.559107	0.011559	0.536452	0.581763	413002	604
Fritsche2018a	0.492231	0.012128	0.46846	0.516002	412399	603
Fritsche2018b	0.489194	0.011584	0.466491	0.511898	412399	603
