# Supplementary material for: The current state of genetic risk models for the development of kidney cancer: a review and validation
Source: BJU Int. 2022 May 7;130(5):550–61. doi: 10.1111/bju.15752 (PMC9790357; doi:10.1111/bju.15752)
Supplement: Supplementary file 4 — Table S8 . Model discrimination (AUROC) in sensitivity analyses. [file BJU-130-550-s002.zip › BJU_15752_TableS8_SA_excluding_all_3rd_degree_relatives.pdf]

model	AUC	AUC_se	AUC_lb	AUC_ub	cohort	cases
Chang2014	0.495145	0.011287	0.473024	0.517267	358627	521
Chen2011a	0.555098	0.011502	0.532554	0.577641	359514	519
Chen2011b	0.530807	0.01117	0.508915	0.5527	359777	519
Chu2012a	0.515397	0.01109	0.493661	0.537133	357269	516
Chu2012b	0.515957	0.011714	0.492999	0.538916	358610	517
Chu2012c	0.508898	0.011155	0.487035	0.530762	358437	519
Coric2016	0.501348	0.011731	0.478356	0.52434	360787	521
DeMartino2016	0.506175	0.013633	0.479454	0.532896	304944	434
Li2012a	0.619973	0.012284	0.595897	0.644048	352555	515
Li2012b	0.621042	0.012237	0.597058	0.645025	352555	515
Li2012c	0.611485	0.0125	0.586985	0.635985	352555	515
Lin2008a	0.494916	0.012419	0.470575	0.519257	360787	521
Lin2008b	0.498842	0.012015	0.475294	0.522391	352708	512
Machiela2017a	0.513103	0.012419	0.488763	0.537443	360787	521
Machiela2017b	0.514172	0.012783	0.489118	0.539226	360787	521
Scelo2016	0.539752	0.012464	0.515323	0.564182	360787	521
Shu2013	0.506339	0.011842	0.483129	0.52955	360787	521
Verma2015	0.515123	0.012434	0.490752	0.539493	355214	514
Wei2014a	0.485666	0.011719	0.462696	0.508635	360787	521
Wei2014b	0.512909	0.010574	0.492185	0.533634	344093	501
Wu2016a	0.503136	0.013035	0.477587	0.528684	360787	521
Wu2016b	0.510243	0.013215	0.484342	0.536144	360787	521
Graff2021	0.540743	0.012584	0.51608	0.565407	360787	521
Shi2019a	0.537717	0.012556	0.513107	0.562327	360787	521
Shi2019b	0.537554	0.012563	0.512931	0.562177	360787	521
Fritsche2021a	0.502496	0.012877	0.477257	0.527734	360207	520
Fritsche2021b	0.502496	0.012877	0.477257	0.527734	360207	520
Kachuri2020	0.53424	0.012608	0.509529	0.558951	360787	521
Jia2020	0.547407	0.012432	0.523041	0.571772	360787	521
Fritsche2018a	0.486065	0.013031	0.460525	0.511605	360207	520
Fritsche2018b	0.492751	0.012687	0.467885	0.517617	360207	520
