# Supplementary material for: The current state of genetic risk models for the development of kidney cancer: a review and validation
Source: BJU Int. 2022 May 7;130(5):550–61. doi: 10.1111/bju.15752 (PMC9790357; doi:10.1111/bju.15752)
Supplement: Supplementary file 4 — Table S8 . Model discrimination (AUROC) in sensitivity analyses. [file BJU-130-550-s002.zip › BJU_15752_TableS8_SA_excluding_multiply_related.pdf]

model	AUC	AUC_se	AUC_lb	AUC_ub	cohort	cases
Chang2014	0.492367	0.010395	0.471994	0.512741	432949	613
Chen2011a	0.556456	0.010551	0.535776	0.577137	433961	612
Chen2011b	0.533515	0.010288	0.513352	0.553678	434279	612
Chu2012a	0.514276	0.01021	0.494264	0.534288	431305	610
Chu2012b	0.51519	0.010802	0.494018	0.536362	432876	610
Chu2012c	0.507613	0.010282	0.487461	0.527766	432638	612
Coric2016	0.492211	0.010784	0.471075	0.513348	435411	615
DeMartino2016	0.505789	0.012454	0.48138	0.530198	368779	516
Li2012a	0.614206	0.011372	0.591917	0.636495	425748	608
Li2012b	0.615512	0.011318	0.593328	0.637696	425748	608
Li2012c	0.608548	0.011503	0.586002	0.631094	425748	608
Lin2008a	0.500128	0.011449	0.477688	0.522568	435411	615
Lin2008b	0.496401	0.011068	0.474708	0.518093	425934	605
Machiela2017a	0.521474	0.01143	0.499072	0.543876	435411	615
Machiela2017b	0.520273	0.011674	0.497392	0.543153	435411	615
Scelo2016	0.550652	0.011545	0.528024	0.57328	435411	615
Shu2013	0.504729	0.010929	0.483309	0.52615	435411	615
Verma2015	0.526319	0.011399	0.503977	0.54866	428726	604
Wei2014a	0.489628	0.010947	0.468172	0.511084	435411	615
Wei2014b	0.510341	0.009795	0.491143	0.529538	415512	593
Wu2016a	0.503909	0.011998	0.480393	0.527425	435411	615
Wu2016b	0.509078	0.012193	0.48518	0.532976	435411	615
Graff2021	0.552849	0.011568	0.530176	0.575521	435411	615
Shi2019a	0.546022	0.011556	0.523373	0.56867	435411	615
Shi2019b	0.545932	0.01156	0.523274	0.568589	435411	615
Fritsche2021a	0.509363	0.011854	0.486131	0.532596	434809	614
Fritsche2021b	0.509363	0.011854	0.486131	0.532596	434809	614
Kachuri2020	0.547179	0.011561	0.524521	0.569838	435411	615
Jia2020	0.559813	0.011436	0.537399	0.582227	435411	615
Fritsche2018a	0.484289	0.011927	0.460912	0.507666	434809	614
Fritsche2018b	0.487752	0.011463	0.465284	0.51022	434809	614
