# Supplementary material for: The current state of genetic risk models for the development of kidney cancer: a review and validation
Source: BJU Int. 2022 May 7;130(5):550–61. doi: 10.1111/bju.15752 (PMC9790357; doi:10.1111/bju.15752)
Supplement: Supplementary file 4 — Table S8 . Model discrimination (AUROC) in sensitivity analyses. [file BJU-130-550-s002.zip › BJU_15752_TableS8_SA_men_only.pdf]

model	AUC	AUC_se	AUC_lb	AUC_ub	cohort	cases
Chang2014	0.488267	0.013169	0.462456	0.514078	198308	392
Chen2011a	0.552797	0.012928	0.527458	0.578135	198773	391
Chen2011b	0.514694	0.012751	0.489702	0.539685	198930	391
Chu2012a	0.517726	0.01288	0.492482	0.54297	197589	390
Chu2012b	0.513509	0.013264	0.487512	0.539506	198309	390
Chu2012c	0.510099	0.01295	0.484718	0.53548	198143	391
Coric2016	0.502569	0.013869	0.475387	0.529751	199423	393
DeMartino2016	0.520187	0.015011	0.490767	0.549607	168927	340
Li2012a	0.61377	0.014243	0.585854	0.641687	194866	389
Li2012b	0.614459	0.014169	0.586687	0.64223	194866	389
Li2012c	0.621913	0.014264	0.593957	0.649869	194866	389
Lin2008a	0.506709	0.01451	0.47827	0.535149	199423	393
Lin2008b	0.500713	0.013866	0.473537	0.527889	195032	388
Machiela2017a	0.503279	0.014264	0.475323	0.531235	199423	393
Machiela2017b	0.49965	0.014688	0.470861	0.528438	199423	393
Scelo2016	0.546868	0.014804	0.517852	0.575884	199423	393
Shu2013	0.50505	0.014017	0.477578	0.532523	199423	393
Verma2015	0.509679	0.01432	0.481612	0.537745	196347	387
Wei2014a	0.481815	0.013775	0.454817	0.508814	199423	393
Wei2014b	0.518172	0.01194	0.49477	0.541575	190188	379
Wu2016a	0.495247	0.014943	0.46596	0.524535	199423	393
Wu2016b	0.50149	0.01508	0.471935	0.531046	199423	393
Graff2021	0.545377	0.014663	0.516638	0.574115	199423	393
Shi2019a	0.53658	0.014973	0.507233	0.565927	199423	393
Shi2019b	0.536414	0.01498	0.507053	0.565774	199423	393
Fritsche2021a	0.506235	0.014964	0.476907	0.535563	199104	392
Fritsche2021b	0.506235	0.014964	0.476907	0.535563	199104	392
Kachuri2020	0.542123	0.014732	0.51325	0.570997	199423	393
Jia2020	0.555628	0.014614	0.526986	0.58427	199423	393
Fritsche2018a	0.4885	0.015164	0.458779	0.518221	199104	392
Fritsche2018b	0.496147	0.014735	0.467268	0.525026	199104	392
