# Supplementary material for: The current state of genetic risk models for the development of kidney cancer: a review and validation
Source: BJU Int. 2022 May 7;130(5):550–61. doi: 10.1111/bju.15752 (PMC9790357; doi:10.1111/bju.15752)
Supplement: Supplementary file 4 — Table S8 . Model discrimination (AUROC) in sensitivity analyses. [file BJU-130-550-s002.zip › BJU_15752_TableS8_SA_women_only.pdf]

model	AUC	AUC_se	AUC_lb	AUC_ub	cohort	cases
Chang2014	0.499173	0.01693	0.465992	0.532355	234801	221
Chen2011a	0.536573	0.01805	0.501197	0.57195	235347	221
Chen2011b	0.54739	0.017633	0.512829	0.581951	235509	221
Chu2012a	0.508095	0.016786	0.475195	0.540996	233876	220
Chu2012b	0.517796	0.018587	0.481367	0.554225	234727	220
Chu2012c	0.502819	0.016936	0.469624	0.536013	234654	221
Coric2016	0.473927	0.017005	0.440598	0.507257	236149	222
DeMartino2016	0.517851	0.021919	0.474891	0.560812	199984	176
Li2012a	0.632694	0.018501	0.596433	0.668954	231037	219
Li2012b	0.630668	0.018512	0.594386	0.66695	231037	219
Li2012c	0.638452	0.018384	0.60242	0.674483	231037	219
Lin2008a	0.488497	0.01862	0.452002	0.524993	236149	222
Lin2008b	0.489098	0.018408	0.45302	0.525176	231060	217
Machiela2017a	0.554009	0.01896	0.516848	0.591171	236149	222
Machiela2017b	0.556812	0.01902	0.519533	0.594091	236149	222
Scelo2016	0.556634	0.018402	0.520568	0.592701	236149	222
Shu2013	0.504097	0.01741	0.469974	0.538221	236149	222
Verma2015	0.556123	0.018713	0.519446	0.592801	232538	217
Wei2014a	0.503204	0.018052	0.467823	0.538585	236149	222
Wei2014b	0.496477	0.017021	0.463116	0.529839	225476	214
Wu2016a	0.519336	0.020157	0.479829	0.558843	236149	222
Wu2016b	0.522352	0.020734	0.481713	0.56299	236149	222
Graff2021	0.565896	0.018821	0.529008	0.602784	236149	222
Shi2019a	0.562767	0.01795	0.527587	0.597947	236149	222
Shi2019b	0.562808	0.017953	0.52762	0.597995	236149	222
Fritsche2021a	0.514902	0.019489	0.476703	0.5531	235865	222
Fritsche2021b	0.514902	0.019489	0.476703	0.5531	235865	222
Kachuri2020	0.556267	0.018622	0.519769	0.592766	236149	222
Jia2020	0.566986	0.018354	0.531013	0.60296	236149	222
Fritsche2018a	0.477337	0.01932	0.43947	0.515203	235865	222
Fritsche2018b	0.472621	0.01813	0.437087	0.508155	235865	222
