# Supplementary material for: The current state of genetic risk models for the development of kidney cancer: a review and validation
Source: BJU Int. 2022 May 7;130(5):550–61. doi: 10.1111/bju.15752 (PMC9790357; doi:10.1111/bju.15752)
Supplement: Supplementary file 5 — Appendix S1 . Supplementary Methods Section. [file BJU-130-550-s005.docx]

**Supplementary Methods Section**

# UKB Cohort

UK Biobank (UKB) was used as the validation cohort in this study. It is the largest population-based cohort in the UK [1]; between 2006 and 2011 more than 500,000 individuals who were aged between 40-69, lived within 25 miles of one of the 22 nationwide assessment centres and were registered with the National Health Service (NHS) were recruited (response rate 5%). All attended a baseline assessment that included completion of questionnaires about lifestyle and medical history and measurement of a range of physical characteristics. Although recruitment for the UKB cohort was designed to be wide reaching, there is evidence of selection bias [2]. The participants differ from the general population in demographics, lifestyle and health outcomes; the rates of cancer are 11.8% lower than in the UK population [2].

Data on cancer incidence is available for UKB participants through linkage to national cancer registries. In this study, kidney cancer was identified with the following diagnosis codes: ICD9 1890 and ICD10 C64 (cancer of the renal pelvis was excluded).

For all UK Biobank participants blood samples were genotyped using Affymetrix UK BiLEVE Axiom Array and Affymetrix UK Biobank Axiom array and imputed to the combined 1000 Genomes Project v.3 and UK10K reference panels using SHAPEIT3 and IMPUTE3. Where possible continuous values for each single nucleotide polymorphisms (SNP) were used (including imputed non-integer values). Categorical SNP variables were created as required, for example, they were needed for models using combined odds ratios to calculate risk, where for each SNPs a different risk was assigned to each combination of alleles (e.g. AA, AT and TT). The SNPs variables were recoded accordingly, 0 – 0.1 => 0, 0.9-1.1=> 1 and 1.9 – 2 => 2, all other values were coded as missing.

Cohort Definition

Members of the cohort with a diagnosis of kidney cancer prior to baseline were excluded from analysis. If more than one diagnosis was recorded, the first occurrence was used. The most recent cancer diagnosis in the dataset is December 2016. We censored all follow-up to 31^st^ March 2016 (the study end date) to ensure that late registrations were not missed. A closed cohort analysis was conducted; individuals whose follow-up was censored before 6 years were excluded. Cases were those who developed kidney cancer within 6-years of baseline assessment.

Model Selection for Validation

Of the identified models (n=39), which predicted the development of kidney cancer and incorporated genetic risk factors, as small number were not included in the validation presented in this paper. This was either due to their use of multiple risk factors not available in UKB, or insufficient information being available to validation those models (authors were contacted in the first instance for additional information).

The models excluded from the validation are:

- Arjumand et al. [3] – insufficient information available to validate these two model
- Hsueh et al. (2017) [4] – both models included only two risk factors, one of which – 8-OHdG and arsenic exposure respectively - is not available for the whole UKB cohort
- Hsueh et al. (2018) [5] – all four models include two risk factors not available for the whole UKB cohort (urinary creatinine and arsenic levels)

Sensitivity Analyses

We carried out several sensitivity analyses, specifically focusing on areas of relevance to genetic risk scores. Firstly, we compared the performance of risk scores after excluding people with high degree of relatedness to other cohort members (people with >10 relatives (n=435411), estimated using genetic data). We also repeated the analysis in a cohort with all one of each pair of relatives (third-degree or closer) randomly excluded (n=360787).

We also compared the performance of the models in the whole cohort, to the performance only in individuals reporting *White/European* ethnicity (n=413002). Given the small numbers of people reporting non-white ethnicity in UKB model performance was not measured for other ethnic groups. We also measured the performance of the models separately for men and women (n = 199,423 and n= 236149 respectively).

Finally, we performed a complete-case analysis using a subset of participants with complete data available for all models (n=163142).

Missing Data

Most of the SNPs used have low levels of missing data, however, some models use SNPs with low imputation scores (lowest imputation score, used in Lin2008 [6], is 0.54). This leads to high levels of missing data if the SNPs are recoded as categorical variables. The imputation score for each SNP in this dataset is given in the table of SNPs used (supplementary materials).

Most of the phenotypic variables used, either in the mixed models or to stratify the cohort in the sensitivity analyses, also had low levels of missing data. All of these variables were drawn from the UKB baseline assessment only. Details of how each variable was used and the levels of missing data are given in Table S4.

One model (DeMartino et al. [7]) used a “pack-years” smoking variable to indicate dose of tobacco exposure. This variable could be calculated by combining information from several smoking variables obtained at the baseline assessment, including smoking start-age, smoking stop-age (for former smokers) and number of cigarettes smoked daily (see Table S4 for details). However, one third of all ever smokers in the UKB cohort have missing data for at least one of the variables required to calculate a measure of pack-years.

Following the method developed by Robbins et al. [8], we first approximated the smoking start-age, for all ever smokers with this value missing, to be 17 years. We then split the cohort into never, former and ever smokers. For the former and current smokers we carried out a chained multiple imputation using STATA’s predictive mean matching method to estimate smoking intensity and for the former smokers, their smoking quit-age (calculated as a percentage of their current age – smoking start-age).

Calibration

Calibration of all included was assessed graphically, the graphs are included in a supplementary file. The expected and the observed risk of developing kidney cancer were compared over the 6-year follow-up period.

We stratified our analysis cohort by deciles of predicted risk; the 5^th^ decile was used as baseline. The observed risk in each decile is the number of cases in that decile, divided by the number of individuals in that decile. The expected risk is the mean risk predicted by the model being assessed for the individuals in that decile. Both the expected and observed relative risk were normalised to their respective values in the 5^th^ decile. If the model has less than 10 values for risk it cannot be split into deciles. Where this is the case, the predicted values are split into the maximum number of possible groups and the median group is used as the baseline instead of the 5^th^ decile.

In all of the calibration plots the line showing ideal calibration (equal observed and expected risk) is plotted. The 95% confidence intervals of the observed risk estimate are also given.

1. Allen, N., Sudlow, C., Downey, P., et al., *UK Biobank: Current status and what it means for epidemiology.* Health Policy Technol, 2012. **1**(3): p. 123-126.

2. Fry, A., Littlejohns, T.J., Sudlow, C., et al., *Comparison of Sociodemographic and Health-Related Characteristics of UK Biobank Participants With Those of the General Population.* Am J Epidemiol, 2017. **186**(9): p. 1026-1034.

3. Arjumand, W., Ahmad, S.T., Seth, A., Saini, A.K., and Sultana, S., *Vitamin D receptor FokI and BsmI gene polymorphism and its association with grade and stage of renal cell carcinoma in North Indian population.* Tumor Biology, 2012. **33**(1): p. 23-31.

4. Hsueh, Y.M., Lin, Y.C., Chen, W.J., et al., *The polymorphism XRCC1 Arg194Trp and 8-hydroxydeoxyguanosine increased susceptibility to arsenic-related renal cell carcinoma.* Toxicology and Applied Pharmacology, 2017. **332**: p. 1-7.

5. Hsueh, Y.M., Chen, W.J., Lin, Y.C., et al., *Adiponectin gene polymorphisms and obesity increase the susceptibility to arsenic-related renal cell carcinoma.* Toxicology and Applied Pharmacology, 2018. **350**: p. 11-20.

6. Lin, J., Pu, X., Wang, W., et al., *Case-control analysis of nucleotide excision repair pathway and the risk of renal cell carcinoma.* Carcinogenesis, 2008. **29**(11): p. 2112-2119.

7. de Martino, M., Taus, C., Lucca, I., et al., *Association of human telomerase reverse transcriptase gene polymorphisms, serum levels, and telomere length with renal cell carcinoma risk and pathology.* Molecular Carcinogenesis, 2016. **55**(10): p. 1458-66.

8. Robbins, H.A., Alcala, K., Swerdlow, A.J., et al., *Comparative performance of lung cancer risk models to define lung screening eligibility in the United Kingdom.* British Journal of Cancer, 2021. **124**(12): p. 2026-2034.
